# Investigating the efficacy of the reminder-extinction procedure to disrupt contextual threat memories in humans using immersive Virtual Reality

**DOI:** 10.1038/s41598-020-73139-4

**Published:** 2020-10-12

**Authors:** Maxime C. Houtekamer, Marloes J. A. G. Henckens, Wayne E. Mackey, Joseph E. Dunsmoor, Judith R. Homberg, Marijn C. W. Kroes

**Affiliations:** 1 Department of Cognitive Neuroscience, Donders Institute for Brain, Cognition, and Behaviour, Radboud University Nijmegen Medical Center, Kapittelweg 29, 6500 HB Nijmegen, The Netherlands; 2 Center for Neural Science, New York University, New York, NY 10003 USA; 3 Department of Psychiatry, University of Texas at Austin, Austin, TX 78712 USA

**Keywords:** Neuroscience, Psychology

## Abstract

Upon reactivation, consolidated memories can enter a temporary labile state and require restabilisation, known as reconsolidation. Interventions during this reconsolidation period can disrupt the reactivated memory. However, it is unclear whether different kinds of memory that depend on distinct brain regions all undergo reconsolidation. Evidence for reconsolidation originates from studies assessing amygdala-dependent memories using cue-conditioning paradigms in rodents, which were subsequently replicated in humans. Whilst studies providing evidence for reconsolidation of hippocampus-dependent memories in rodents have predominantly used context conditioning paradigms, studies in humans have used completely different paradigms such as tests for wordlists or stories. Here our objective was to bridge this paradigm gap between rodent and human studies probing reconsolidation of hippocampus-dependent memories. We modified a recently developed immersive Virtual Reality paradigm to test in humans whether contextual threat-conditioned memories can be disrupted by a reminder-extinction procedure that putatively targets reconsolidation. In contrast to our hypothesis, we found comparable recovery of contextual conditioned threat responses, and comparable retention of subjective measures of threat memory, episodic memory and exploration behaviour between the reminder-extinction and standard extinction groups. Our result provide no evidence that a reminder before extinction can prevent the return of context conditioned threat memories in humans.

## Introduction

A brief reminder can return consolidated memories to a labile state, requiring re-stabilization processes to maintain the memory, a process referred to as reconsolidation^1,2^, but see^3–5^ for alternative accounts. Interventions that target reconsolidation can modify long-term memories^1^. This discovery has led to suggestions that reconsolidation-targeting interventions might be used to modify maladaptive memories as a treatment for stress- and anxiety-related disorders^6–10^. Yet another important realization of memory research is that there are distinct kinds of memory that rely on different brain regions and are expressed in different forms of behaviour^11–13^. People with stress- and anxiety-disorders generally experience several different forms of maladaptive memory expression, such as excessive threat responses, subjective negative feelings, emotional episodic memories, and avoidance behaviours^14–17^. Critically, to date it is still unclear whether all kinds of memory undergo reconsolidation and whether these are equally sensitive to reconsolidation-targeting interventions. Here, we test whether contextual threat conditioned memories are sensitive to disruption through a behavioural reconsolidation-targeting intervention.

Studies using simple cue conditioning paradigms, in which e.g. a single tone predicts a shock, have provided evidence for reconsolidation in both rodents^1^ and humans^18^ (for a review, see^2,8^). In Pavlovian cue conditioning, the formation and storage of the mnemonic association between the conditioned stimulus (CS, e.g. a tone) and the unconditioned stimulus (US, e.g. a shock) is amygdala-dependent (for a review, see Ref.^19^). Unlike the highly controlled cued threat-memory paradigms used in laboratory settings, real-life emotional memories can also include information about the spatiotemporal context of a threatening experience, and are more hippocampus-dependent^20–25^. Therefore, to understand the implications and limitations of reconsolidation interventions for the potential treatment of stress- and anxiety-disorders, it is imperative to also know the impact of interventions targeting the putative reconsolidation process on forms of threat memories that are primarily hippocampus-dependent

In rodents, evidence for reconsolidation of hippocampus-dependent memories has been obtained using Pavlovian contextual threat conditioning paradigms, where animals learn an association between a particular contextual environment (i.e. the conditioning chamber) and an aversive outcome (i.e. a shock) in the absence of a discrete cue signalling the outcome^26–30^. In humans, however, supportive evidence for reconsolidation of hippocampus-dependent memories is more limited and in contrast to studies in rodents generally stems from outside the Pavlovian threat conditioning domain, relying on memory paradigms originating from the episodic memory domain, such as word-lists^31,32^ or stories^10,33–35^. A recent study trying to unite approaches from the Pavlovian threat conditioning and episodic memory fields, using a category threat conditioning procedure in humans, indicated that episodic memory for items that were part of a Pavlovian threat conditioning experience can undergo reconsolidation, but that the efficacy of reconsolidation-interventions may decrease when episodic memory demands increase^36^, consistent with suggestions from studies with rodents^37^ (see Ref.^8^ for a review). Although category conditioning involves hippocampal processing^38,39^, the paradigm is still quite different from reconsolidation studies with rodents that have been able to directly interfere with hippocampal processing using contextual conditioning. To further close the gap between rodent and human studies it would be useful to test for the reconsolidation of contextual threat conditioned memories in humans.

Recently, an immersive Virtual Reality (iVR) contextual threat conditioning paradigm for humans was developed, which provides people with a sense of immersion in a virtual environment and allows people to learn an association between a particular contextual environment and an aversive outcome in the absence of a discrete cue signalling the outcome. This iVR context conditioning paradigm was shown to result in the acquisition of contextual threat-conditioned defensive responses, subjective feelings of threat, and episodic memory for details of the threatening spatiotemporal context^40^. As similar VR contextual threat conditioning paradigms have been shown to involve hippocampal processing in humans (Ref.^41^ and see Ref.^40^ for a review), this iVR contextual threat conditioning paradigm provides an opportunity to investigate reconsolidation of contextual threat conditioned memories that are likely hippocampus-dependent and comparable to memories in studies using contextual threat conditioning procedures in rodents.

To interfere with reconsolidation, the majority of studies have used pharmacological interventions (for a review, see Refs.^2,8^). Yet behavioural interventions, such as the reminder-extinction procedure, may also be able to influence reconsolidation of memory^42^. This is an exciting discovery as behavioural interventions can be considered preferable as they are inherently more accessible and safe compared to pharmacological interventions^43,44^. In the behavioural reminder-extinction procedure an isolated reminder of a threat memory is presented to return the memory to a labile state and next (typically after 10 min) standard extinction training is performed. The reminder-extinction procedure has been found to persistently attenuate cued threat memories in both rodents and humans^45–54^ (yet for non-replications see e.g. Refs.^55–62^ and see Ref.^34^ for a meta-analysis). Studies showing that the reminder-extinction procedure can prevent the return of threat responses have postulated that the reminder triggers a reconsolidation process, and extinction training during this reconsolidation window can overwrite the original threat memory^42^. Whether indeed the reminder-extinction depends specifically on disruption of the original memory remains to be determined^63^, and alternative explanations for the efficacy of the reminder-extinction procedures include active memory integration accounts^4^ and the enhanced-extinction account^64^.

Presenting an isolated reminder before extinction has previously shown to enhance attenuation of contextual conditioned threat responses in mice^65^ and rats^27,66–68^ (yet for non-replications, see^69,70^). In humans, however, an indirect translation of the contextual conditioning paradigm, using compound stimuli consisting of fear-relevant cues presented in different frames to represent different contexts, suggested that the reminder-extinction paradigm does not prevent spontaneous recovery of threat responses^71^. In addition, the above mentioned category threat conditioning study^36^ indicated that the efficacy of the reminder-extinction procedure might be limited when episodic memory demands increased. We therefore wondered whether we could reproduce previous findings in rodents^27,65^, showing attenuation of contextual threat responses after presentation of a reminder before extinction, in humans using a direct translation of rodent contextual threat conditioning paradigms.

**Figure 1:**
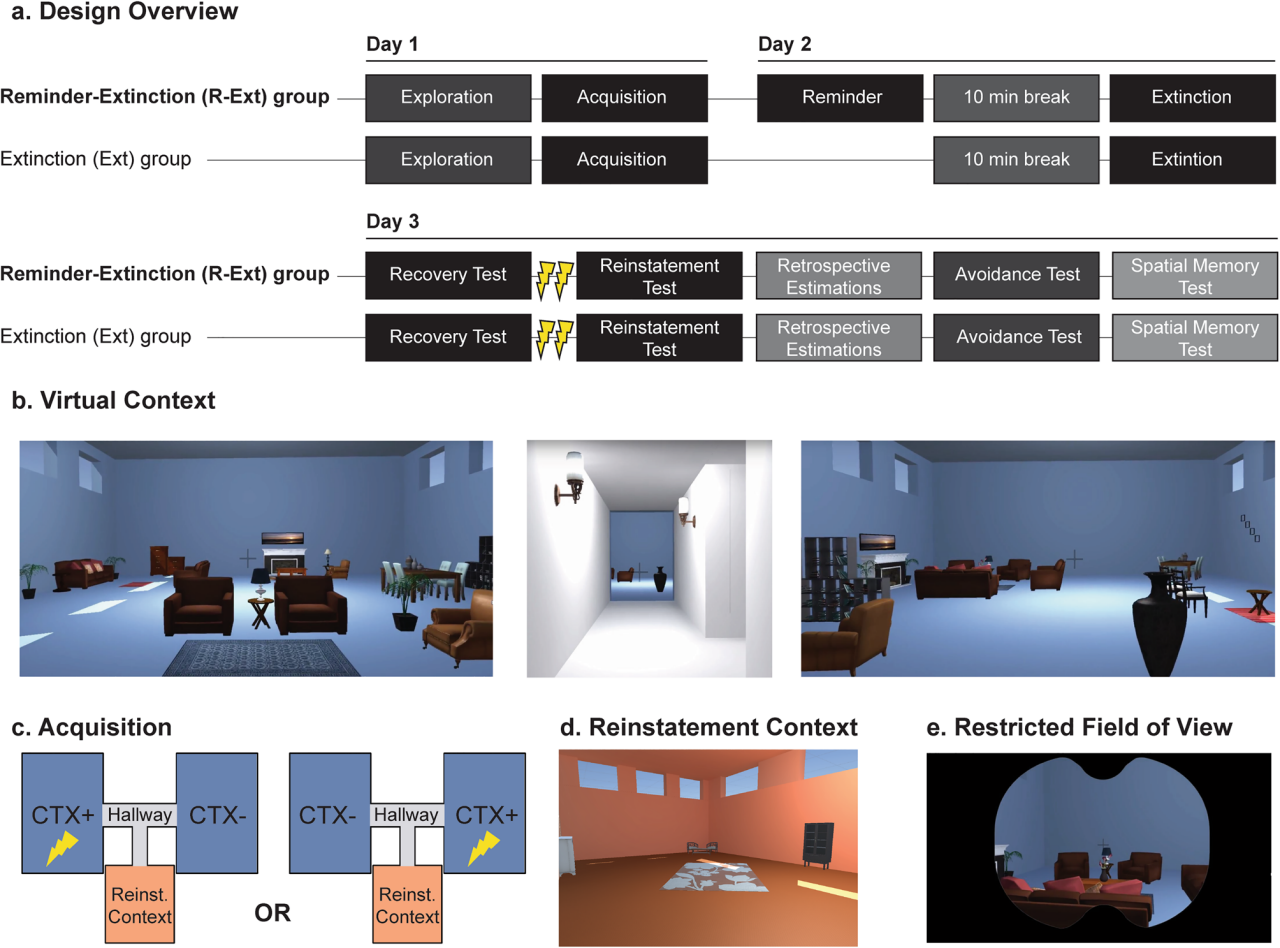
Three-day between-subjects iVR contextual threat conditioning study design. (**a**) Time-line of the experimental design, displayed separately for the Reminder-Extinction (R-Ext) group and the Extinction (Ext) group. Task in the darkest hues are carried out in iVR (exploration, acquisition, reminder, extinction, recovery test and reinstatement test). (**b**) 2D depiction of the two blue rooms and the connecting hallway in the iVR environment. (**c**) Schematic depiction of the iVR context, as seen from above. The two blue rooms are the conditioned context (CTX+, coupled with shocks) and safe context (CTX−, never coupled with shocks), counterbalanced between participants. The hallway (displayed in light grey) connects the two blue rooms, and a third, orange room was used for reinstatement (Reinst. Context). (**d**) 2D depiction of the orange room in which participants received shocks for reinstatement on day 3. (**e**) To minimize potential motion sickness, the field of view was dynamically restricted: on straight paths, participants had a wide field of view, and on sharp turns, the field of view was restricted as displayed in the picture.

Therefore, the objective of this preregistered study (https://osf.io/b2854/) was to investigate the efficacy of the reminder-extinction procedure to prevent the return of contextual threat conditioned memories in humans. To achieve this, participants (N = 60)—in a between-subjects design—navigated through an immersive Virtual Reality (iVR) environment where they received aversive electrical shocks to create, modify, and test contextual threat-conditioned memory in a controlled laboratory setting (see Fig. 1a–e, a modification of the tasks used in^40^). In brief, on day 1 participants were differentially conditioned to a context signalling threat (CTX+) of receiving a transcutaneous electrical shock (US) and a safe context (CTX−). On day 2, participants in one group (reminder-extinction group) were presented with an isolated reminder of the conditioned context (CTX+), while the other group was not (extinction group). After a 10-min break, both groups underwent extinction training. On day 3, we tested for the return of context-conditioned threat responses, subjective threat memory, contextual avoidance, and episodic memory in both groups. We hypothesized that the reminder-extinction group would show attenuated recovery of contextual threat conditioned responses, may exhibit reduced avoidance of the threatening context, and potentially altered episodic memory. We followed our preregistered design and analyses with a few minor exceptions, which we clearly indicate below. In contrast to our hypotheses, yet in line with previous non-replications for cue conditioned threat memories^55,56,58–61,71–73^, contextual conditioned threat memories^71^ and category threat conditioned memories^36^, we found comparable recovery of context conditioned threat responses in the extinction group and the reminder-extinction group, and no group differences on either avoidance behaviour or the other memory tests, suggesting that the reminder-extinction procedure did not modify contextual threat memories in humans.

## Methods

### Participants

Sixty healthy volunteers (40 female, 20 male; 18–30 years [22.21 ± 0.40]) completed the study. Twenty-six additional participants signed up but did not complete the study: 13 failed to attend all three experimental sessions and 13 had to be discarded due to apparatus failure. Among subjects excluded due to apparatus failure, 9 participants were excluded because the VR equipment disconnected which interrupted the task so that participants no longer saw anything through the goggles. Two participants were excluded due to malfunction of the shock equipment where they did not receive any shocks and two were excluded due to a broken fiber optic cable due to which the physiological data did not contain any event markers. Participants enrolled in the study through a local online psychology research website (SONA) and were fluent in Dutch. Exclusion criteria were: current or lifetime history of psychiatric, neurological, or endocrine illness, abnormal hearing or (uncorrected) vision, average use of more than 3 alcoholic beverages daily, current treatment with any medication that affects central nervous system or endocrine systems, average use of recreational drugs weekly or more, predominant left-handedness (to prevent potential differences in threat responses between left and right handed participants) and proneness to motion sickness. All participants provided written informed consent and received 35 Euro monetary compensation for their participation. As an additional incentive, participants could receive an additional monetary compensation of 5 Euros if they correctly answered 70% of questions on a spatial memory test at the end of the study. The study was approved by the local ethical review board (CMO region Arnhem-Nijmegen). All participants provided written informed consent. All methods were carried out in accordance with the Declaration of Helsinki.

### Immersive Virtual Reality environment

A Virtual Reality environment was designed in Unity 5 (Unity Technologies, https://www.unity3d.com), based on a previously used paradigm^40^. The environment consisted of three virtual living rooms connected via a hallway. Two rooms had blue flooring and walls, and contained identical items, and a third room had orange walls and flooring and contained different living room decorations (see Fig. 1b). Note, we made a critical modification to the previous version of this iVR context conditioning paradigm^40^ to theoretically increase the necessity for hippocampal processing, where now the CTX+ and CTX− rooms had the same colour and contained the same furniture items and could only be distinguished based on the arrangement of the furniture items relative to each other, and their location in space relative to the orange room. To minimize potential discomfort or nausea due to the movement in iVR, a static fixation cross was presented in the middle of the screen, and the field of view was dynamically controlled to be minimal during sharp turns and maximal on straight paths (see Fig. 1e). In addition, an opaque red line projected just above the floor displayed the path ahead.

### Contextual threat conditioning task

During all contextual threat conditioning iVR tasks, participants passively navigated through two blue rooms and the hallway on pre-defined paths. During the threat acquisition task, visits to one blue room were paired with shocks (CTX+) but not in the other blue room (CTX−) or the hallway (see Fig. 1c). The electrical shock was a 2 ms pulse to the distal phalanges of the second and third digit of the right hand using gelled electrodes connected to a constant current stimulator (Digitimer, DS7A; Hertforshire, United Kingdom). To measure conditioned learning, threat potentiation of the eye-blink amplitude was measured in response to loud startle probes presented throughout the task^40^. Startle probes and US occurred pseudo-randomly from 5–25 s after entering a room with the limitation that there had to be 5 s between each event, i.e. between each occurrence of shocks and startle probes. Noise probes in the hallway occurred 5–10 s after entry. During the task of approximately 15 min, each blue room was visited ten times, each visit lasting approximately 30 s. Visits to the CTX+ and CTX− were separated by a 15-s transition through the hallway connecting the two contexts. Six out of ten visits to the conditioned context (CTX+) were paired with one or two shocks (60% reinforcement rate), amounting to a total of eight shocks. The reminder task consisted of a single (30 s) visit to the CTX+ under extinction conditions (i.e., no shock was administered), starting and ending in the hallway, with a total duration of approximately 1 min. We opted for a single reminder trial of 30 s because single reminder trials have been showed to labilize memory whilst more trials trigger extinction learning mechanisms instead^74,75^. We elected for the reminder trial to be as long as an acquisition trial (30 s), as is standard in reconsolidation-targeting cue-conditioning paradigms^1,18,46,76^. The duration of our reminder trial was therefore slightly shorter than the 90 s to 5 min reminders of studies in rodents showing diminishment of contextual conditioned threat responses following a reminder-intervention strategy^65,77–79^ but not as long as long as the 30 min exposure that induce extinction in these studies^65,77,78^. As our result indicate (see below) our single 30 s reminder was long enough to reactivate memory whilst brief enough not to result in extinction learning. The extinction task was of equal duration and set-up as the acquisition task. Extinction consisted of ten visits to the CTX+ and ten visits to the CTX− of approximately 30 s interleaved by twenty 15-s visits to the hallway while no shocks were administered throughout the extinction task, with a total duration of approximately 15 min. It should be noted that we accidently did not adapt the number of CTX+ visits during extinction training for the R-Ext group to compensate for the reminder visit. Thus, including the reminder, the R-Ext group was exposed to one additional CTX+ visit under extinction conditions. To test for spontaneous recovery of the threat response, a shorter version of the task was used with three 30-s visits to each blue room interleaved with six 15-s hallway visits, with a total duration of approximately 5 min. No shocks were administered. To test for the reinstatement of conditioned fear responses, participants were passively guided through the third, orange room (see Fig. 1d) where they received two un-signalled shocks. Afterwards, they were again guided through the two blue rooms, for three 30-s visits to each blue room interleaved with six 15-s visits to the hallway, totalling to approximately 6 min.

### Physiology collection and data analysis

#### Eye-blink startle

Startle responses were measured using electromyography (EMG) of the right orbicularis muscle and evoked using startle probes (binaural bursts of 100 dB white noise presented for 50 ms). Data were collected using a BrainAmp system, recorded with the BrainVision recorder software (Brain Products GmbH, Munich, Germany) and analysed by means of an in-house analysis program written in Matlab (the MathWorks) that uses the FieldTrip toolbox^80^ (as before in^40^). Responses to the startle probe were found to be consistently delayed compared to latencies in previous studies^40,81,82^, due to a 120 ms delay in tone presentation within the iVR task in our current set-up. Therefore, we deviated slightly from our preregistration and, based on the observed mean latency of startle responses across all conditions and participants, determined responses to the startle probe as maximum EMG response between 140 and 240 ms relative to our trial onset marker. A baseline measure of the mean EMG magnitude in a 500 ms window prior to trial onset was subtracted from the maximum EMG response. In line with previous studies, startle responses for each trial were transformed to T-scores (z-score × 10 + 50) for each participant and task separately^40,82,83^.

#### Skin conductance

Electrodermal activity (EDA) was assed using two Ag/AgCl electrodes attached to the distal phalanges of the second and third digit of the left hand. Data were collected using a BrainAmp system and recorded using BrainVision recorder software (Brain Products GmbH, Munich, Germany) and analyzed using an in-house analysis program written in Matlab (the MathWorks) using FieldTrip^80^ (as before in^36,40^). Skin conductance responses (SCRs) were measured after startle bursts and during transitions from the neutral hallway to the conditioned contexts (blue rooms). Responses were defined as the through-to-peak amplitude difference in skin conductance of the largest deflection in the latency window from 0–4.9 s after event onset to ensure that responses could not be contaminated by other events (shocks or following startle probes). The raw skin conductance responses were square root transformed, in line with previous studies^83–85^.

#### Heart rate

Raw pulse data were measured using a pulse oximeter and collected using a BrainAmp system and recorded using BrainVision recorder software (Brain Products GmbH, Munich, Germany). Pulse data were processed offline using in-house software to detect R-peaks automatically, following previous literature^86^. All R-peak time-courses were visually inspected and faulty peak locations were manually corrected. Interbeat intervals, the time between two R-peaks, were calculated, converted to beats per minute (BPM), and down-sampled to 2 Hz. Heart-rate responses were defined as time-series from 0–4 s after event onset expressed as change in BPM with respect to a mean baseline during the 1 s before event onset. Average heart rate (HR) responses were calculated for each stimulus (CTX+, CTX−) per phase of each task (early: first half of trials, late: second half of trials) for each participant.

#### Valence and arousal ratings

Valence and arousal ratings were obtained using self-assessment manikin scales. The valence scale ranged from 1 (= extremely negative) to 10 (= extremely positive). The arousal scale ranged from 1 (= extremely calm) to 10 (= extremely excited).

#### Retrospective shock estimation and contingency awareness questionnaire

The retrospective shock estimation and contingency awareness questionnaire asked participants to estimate the number of shocks they thought they had received and estimate the percentage of times that they had received a shock in each of the blue rooms for each experimental task^40,87^.

#### Avoidance test

In the avoidance task, participants freely navigated through the two blue rooms and the hallway in search of a hidden coin reflecting a monetary reward for 2 min. Their location was continuously monitored. In reality no coins were present anywhere and the task was stopped after 2 min, allowing investigation of an equal amount of exploration time and avoidance for each participant. Behaviour was scored as the first room that was visited (CTX+ or CTX−) and the time spent in the CTX+ and the CTX−.

#### Spatial memory test

The spatial memory test consisted of 8 questions probing the position of furniture items in each of the two blue rooms. Participants placed images of furniture items that had been present in the rooms on a spatial grid representation of each room.

#### iVR experience questionnaire

The iVR experience questionnaire assessed on a 5-item scale how participants had felt during the virtual reality tasks (“I felt no discomfort”, “I was a tiny bit uncomfortable, but not too bad”, “I was slightly uncomfortable”, “I was moderately uncomfortable and slightly nauseous”, “I was very uncomfortable and very nauseous”), and whether they had experience using Virtual Reality technology (“No experience” , “Once, a couple of minutes”, “Once for a while”, “For a while on several occasions”, “regularly”) and playing video games in general (“No experience”, “Very limited experience, I hardly ever play video games”, “Nowadays I rarely play video games, but I used to play video games often” , “Regularly”, “Often”)^40^.

#### Inventories and anxiety questionnaires

Participants completed the State-Trait Anxiety Inventory^88^, Intolerance of Uncertainty Scale^89^, Berkman–Syme Social Network Index^90^, and Childhood Trauma Questionnaire—short form^91^. These were included not for the purpose of the current experiment but for an independent investigating individual differences in threat learning over multiple studies.

### Procedures

The design of this study is illustrated in Fig. 1. Participants were pseudorandomly assigned to either the Reminder-Extinction (R-Ext) or Extinction (Ext) group. The study was conducted over three consecutive days. On the first day of the experiment, shocks were calibrated using an ascending staircase procedure starting with a low voltage setting near a perceptible threshold and increasing to a level deemed “maximally uncomfortable but not painful” by the participant, in keeping with prior threat conditioning protocols^40,92,93^.

Participants wore the consumer version of the Oculus Rift headset as previously described^40^. The headphone component of the Oculus rift was removed and replaced by Sennheiser HD 202 (Wedemark, Germany) headphones. Before the acquisition of contextual fear, participants were asked to freely explore the rooms and hallway for 2 min to encourage pre-exposure to the contexts prior to conditioning. After the exploration, valence and arousal ratings were obtained for the different rooms. Next, participants were given a surprise memory test and asked to locate three items in both rooms. After having completed the test, participants were told that they would be asked to complete a similar test on the third day of the experiment, and they were able to receive an additional monetary compensation if they correctly answered at least 10 out of the 16 questions on that test. This spatial memory test and these instructions were added to ensure that participants would pay attention to, and remember, the differences in spatial layout between the two rooms.

Next, participants were equipped with measurement devices for startle response, skin conductance and heart rate. We explained that loud noises would be presented during the next virtual reality task, but that we would start with a brief task to allow the participants to habituate to the sound. Participants listened to 9 startle probes while viewing a blank grey screen (without the VR headset) to allow startle responses to habituate.

After habituation, participants were prepared for the acquisition task, and instructed that they would be visiting the two blue rooms and the hallway, and told to pay attention to the fact that a relationship existed between the two blue rooms and the shocks. The participants were told that they could not receive shocks in the hallway between the rooms. During the threat acquisition task, shocks were administered in one blue room (CTX+) but not in the other blue room (CTX−) or the hallway (see Fig. 1c). After this task, the VR headset was removed and valence and arousal ratings were obtained for the different rooms. Next, recording equipment was removed and participants were thanked for their effort during the session.

Participants returned to the lab the following day, and were immediately equipped with recording devices for startle response, skin conductance and heart rate. Participants that had been assigned to the reminder-extinction group were told that they would again be visiting the different rooms and may receive shocks, and that the task would continue as before. To reactivate the contextual threat conditioned memory, they were guided through the CTX+ once. In line with previous studies, the reminder was followed by a 10 min break, so that the following extinction task would fall within the putative reconsolidation window. During this break, all participants (both the R-Ext as Ext groups) watched 10 min of landscape scenes from BBC Planet Earth (2006 TV series). Participants were explicitly told that they would not receive any shocks during this break, and the shock equipment was visibly turned off for the duration of the break. All participants were then told that the task would continue as before, that they would again hear sounds and might receive shocks. This procedure is in line with previous reports^36,46,52^. Participants where then subjected to the extinction task. After the task, the VR headset was removed and valence and arousal ratings were obtained for the different rooms. Recording equipment was removed and participants were thanked for their efforts.

Participants returned to the lab again the following day for a third session. Startle response, skin conductance and heart rate recording equipment was attached, and participants were instructed that the tasks would continue as before, except for the fact that there would be two shorter tasks immediately following each other. They were told that they would visit the different rooms and could receive shocks. Participants completed the spontaneous recovery task. After the spontaneous recovery task, the reinstatement task was started immediately. To test for the reinstatement of contextual threat conditioned responses, participants were passively guided to the third, orange room (see Fig. 1d), and received two un-signalled shocks while moving through the orange room. Afterwards, the participants were again guided through the two blue rooms and responses to startle probes were measured.

Throughout all tasks in iVR, the participants were attached to the shock electrodes, the shock stimulator was set to the ‘On’ position and they were instructed that they could receive shocks.

After the end of the reinstatement task, valence and arousal ratings were obtained for the different rooms. In addition, participants were asked to estimate the number of shocks they thought they had received and the percentage of times that they received a shock in each of the blue rooms for each experimental task. Next, the participants completed the spatial memory test at their own pace.

Once they completed the memory task, participants were instructed that a coin was hidden in one of the two blue rooms, and that they had 2 min to find the coin. They were told that the task would end automatically if they walked to the coin in iVR. Using the oculus touch controllers, the participants navigated freely for 2 min, after which the task was stopped and the participants were debriefed about the nature of the task.

Participants completed the State-Trait Anxiety Inventory, Intolerance of Uncertainty Scale, Berkman–Syme Social Network Index, and Childhood Trauma Questionnaire—short form at their own pace. We debriefed participants about the purpose of the study and provided information about reimbursement. Finally, participants were given the opportunity to ask questions.

### Statistics

Statistical analyses were performed in SPSS (IBM SPSS Statistics Inc.). Dependent measures were submitted to repeated measure ANOVAs and statistics were Greenhouse–Geisser or Huyn-Feldt corrected for non-sphericity when appropriate (i.e., if sphericity assumptions were violated and epsilon was smaller or greater than 0.75, respectively). Significant findings from ANOVAs were followed-up by paired- and independent samples *t* tests. We report partial eta-square as measure of effect size. Means ± s.e.m are provided where relevant unless otherwise indicated.

## Results

### Participants

We first assessed whether there were any group differences in age, sex, motion sickness during the iVR tasks, experience with iVR game experience and time spent playing games. Exploratory *t* test did not reveal any group differences (All P’s > 0.075). The median response across groups to the iVR question about how participants experienced the iVR was “I was a tiny bit uncomfortable, but not too bad”, and no participants indicated to have felt “[…] very uncomfortable and very nauseous”.

Immediately following the acquisition of contextual threat conditioning, fifty-seven out of sixty participants could explicitly state the relationship between the conditioned contexts and shocks, indicating that they learned the conditioned association. We therefore opted to include as many people as possible for our different dependent measures whilst adhering to our preregistered inclusion criteria. We describe our inclusion criteria and number of included participants for each measure below.

### Fear-potentiated startle

**Figure 2:**
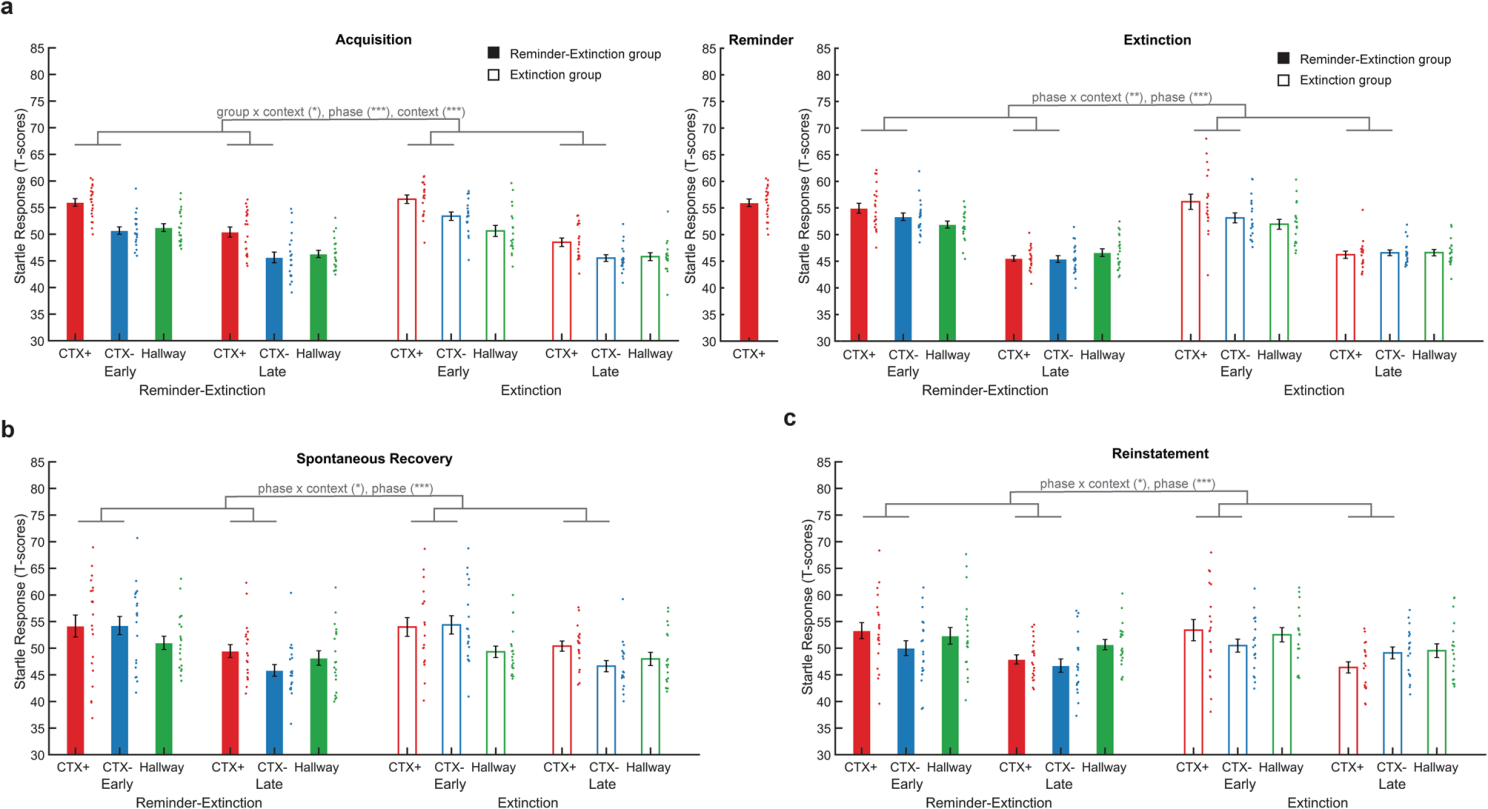
Results of fear-potentiated startle (FPS) response. The contextual threat conditioning procedure resulted in acquisition, retention and extinction of threat-related FPS responses, but an isolated reminder before extinction did not prevent the return of FPS responses on the following day. Bars reflect mean t-scored startle responses during the early (first half of trials) and late phase (second half of trials) of each task for the threat (CTX+, red) and safe context (CTX−, blue) and neutral hallway (green) for the Reminder-Extinction group (solid bars) and Extinction group (open bars). Error bars = s.e.m., adjacent dots represent jittered individual data-points. *p < 0.05, **p < 0.01, ***p < 0.001. (**a**) Both groups acquired comparable differential contextual threat conditioned FPS responses. On day 2, both groups showed comparable extinction of FPS responses, where differential FPS responses were fully extinguished at the end of the task. (**b**) Both groups showed comparable generalized spontaneous recovery of FPS responses to both the CTX+ and CTX− during the early phase of the spontaneous recovery test. Although the initial recovery is generalized, FPS responses in the CTX+ showed slower re-extinction, indicating differential retention of the conditioned FPS responses. (**c**) Following two unsignaled shocks, FPS responses showed evidence for reinstatement as differential responses to the CTX+ and CTX− are greater during the early phase as compared to the late phase.

Fear-potentiated startle (FPS) served as our main index of contextual threat acquisition, reactivation, extinction, and recovery (Fig. 2). As determined in our pre-registration, we only included participants who showed successful conditioning during the acquisition phases, as measured by a numerically greater startle response in the threat (CTX+) compared to the safe context (CTX−). Twenty-one participants (out of twenty-seven) showed numerically greater startle responses in the CTX+ as compared to the CTX− for the R-Ext group, and nineteen (out of thirty-three) participants for the Ext group. These participants were included in further analysis of FPS responses. A complete description of results used to verify comparable acquisition, extinction and retention of contextual conditioned startle responses is included in the supplementary information. Here, for readability, complete statistics are only provided for the critical tests on the return of threat.

During the late phase of the acquisition phase on day 1, we observed comparable discriminatory contextual threat conditioned startle responses between both groups (Fig. 2a). Unexpectedly, over the entire acquisition phase, we observed greater differential FPS responses for the R-Ext than Ext group (t(38) = 2.244, p = 0.031, R-Ext: 5.0 ± 0.70, Ext: 3.0 ± 0.50). However, separate repeated measures ANOVAs (rmANOVAs) for the early and late phase of acquisition showed that at the start of acquisition, there was a trend interaction effect of group × context (F_1,38_ = 53.311, p = 0.077, η^2^ = 0.080) but this trend did not persist during the late phase of acquisition (F_1,38_ = 1.643, p = 0.208, η^2^ = 0.041). Thus, critically, in the late phase of acquisition, both groups showed comparable differences between startle responses in the CTX+ and CTX−, indicating comparable acquisition of contextual conditioned threat responses. In the R-Ext group, the reminder resulted in reactivation of the contextual threat conditioned memory, shown by greater startle responses in the CTX+ than hallway (t(20) = 3.114, p = 0.005, CTX+: 61.8 ± 2.8, hallway: 49.26 ± 1.8, as participants did not traverse the CTX− during the reminder, a comparison between FSP in the CTX+ and CTX− was not possible). In addition, the reminder trial did not trigger extinction learning, indicated by the absence of a reduction in freezing scores from the reminder trial to the first CTX+ trial during extinction (p = 0.775). Afterwards, both groups underwent successful extinction of contextual threat conditioned FPS, which was preceded by an isolated reminder for the R-Ext group. A group (R-Ext, Ext) × phase (early, late) × context (CTX+, CTX−) rmANOVA on FPS responses during the extinction task revealed an interaction of phase × context (F_1,37_ = 8.552, p = 0.006, η^2^ = 0.188) and a main effect of phase (F_1,37_ = 217.726, p < 0.001, η^2^ = 0.855), with no other main effects or interactions. During the late phase of the extinction task, both groups show comparable and successful extinction, indicated by an absence of differential FPS in the late phase of extinction (t(39) = − 0.393, p = 0.696, CTX+: 45.6 ± 0.32, CTX−: 45.8 ± 0.37) that was not significantly different across groups (all Ps > 0.16).

On day three, spontaneous recovery of FPS was tested under extinction conditions. We observed comparable spontaneous recovery of FPS responses in both groups (Fig. 2b). A group (R-Ext, Ext) × phase (early, late) × context (CTX+, CTX−) rmANOVA revealed a significant interaction effect of phase × context (F_1,38_ = 4.452, p = 0.041, η^2^ = 0.105), and a main effect of phase (F_1,38_ = 27.113, p < 0.001, η^2^ = 0.416). Importantly, there was no significant group × context × phase interaction (p = 0.905) or other interaction with or main effects of group (all P’s > 0.5), indicating that spontaneous recovery was not affected by the presentation of an isolated reminder before extinction the previous day. Follow up paired *t* tests revealed greater differential responses (CTX+ – CTX−) in the late compared to the early phase (t(39) = − 2.134, p = 0.039, early: 0.24 ± 1.8, late: 3.7 ± 1.1), which was driven by greater responses in the CTX+ than the CTX− in the late phase (t(39) = 3.264, p = 0.002, CTX+: 49.9 ± 0.74, CTX−: 46.2 ± 0.72) but not in the early phase (t(39) = − 0.133, p = 0.895, CTX+: 54.1 ± 1.4, CTX−: 54.3 ± 1.2). These findings seem to indicate participants across both groups initially showed generalized recovery of threat responses in both the CTX+ and CTX− and over the course of the spontaneous recovery test were slower to extinguish FPS responses in the CTX+ compared to the CTX−, indicating retention of the differential conditioned contextual threat response. To further test for the presence of spontaneous threat recovery, we assessed the change in FPS from the end of extinction to the beginning of spontaneous recovery. A group (R-Ext, Ext) × task (late extinction, early spontaneous recovery) × context (CTX+, CTX−) rmANOVA revealed a main effect of task (F_1,37_ = 79.681, p < 0.001, η^2^ = 0.683) and no significant main effects or interactions of group and or context (all Ps > 0.6). As we found no effect of group or context and an overall change in FPS from one task to another is logically expected for within day T-transformed data we did not follow up on this finding any further.

Next we tested for reinstatement of contextual threat conditioned FPS responses. We found comparable reinstatement of differential FPS responses for both groups (Fig. 2c). A group (R-Ext, Ext) × phase (early, late) × context (CTX+, CTX−) rmANOVA revealed an interaction effect of phase × context (F_1,38_ = 6.077, p = 0.018, η^2^ = 0.138) and a significant main effect of phase (F_1,38_ = 17.334, p < 0.001, η^2^ = 0.313), but no significant interactions with or main effect of group (all P’s > 0.2). A follow up *t* test revealed greater differential responses to the CTX+ and CTX− in the early compared to the late phase of reinstatement (t(39) = 2.405, p = 0.021, early: 3.1 ± 1.4, late: − 0.68 ± 1.1). Specifically, we observed greater startle responses in the CTX+ as compared to the CTX− in the early phase (t(39) = 2.142, p = 0.039, CTX+: 53.3 ± 1.2, CTX−: 50.2 ± 0.89), but not in the late phase (t(39) = − 0.628, p = 0.534, CTX+: 47.2 ± 0.64, CTX−: 47.9 ± 0.82). To test for the increase in responses, mean startle responses were subjected to a task (late recovery test, early reinstatement test) × context (CTX+, CTX−) × group (R-Ext, Ext) rmANOVA. There was a significant main effect of context (F_1,38_ = 12.088, p = 0.001, η^2^ = 0.241) and task (F_1,38_ = 16.426, p < 0.001, η^2^ = 0.302) and no significant main effect of group or interactions with group (all P’s > 0.5). Follow up *t* tests revealed that startle responses were higher during the reinstatement test than during the spontaneous recovery test (t(39) = 4.122, p < 0.001, late spontaneous recovery: 48.1 ± 0.47, early reinstatement: 52.8 ± 0.75), and FPS responses were greater in the CTX+ than in the CTX− (t(39) = 3.53, p = 0.001, CTX+: 51.6 ± 0.76, CTX−: 48.2 ± 0.51). As reinstated responses often extinguish rapidly, we also submitted reinstatement index scores (first trial of reinstatement test − last trial of recovery test) to a context (CTX+, CTX−) × group (R-Ext, Ext) rmANOVA. There were no significant main or interaction effects of group and context (all P’s > 0.3). This suggests that initial reinstatement generalizes to both the CTX+ and CTX−, and is not affected by a reminder.

### Valence and arousal

**Figure 3:**
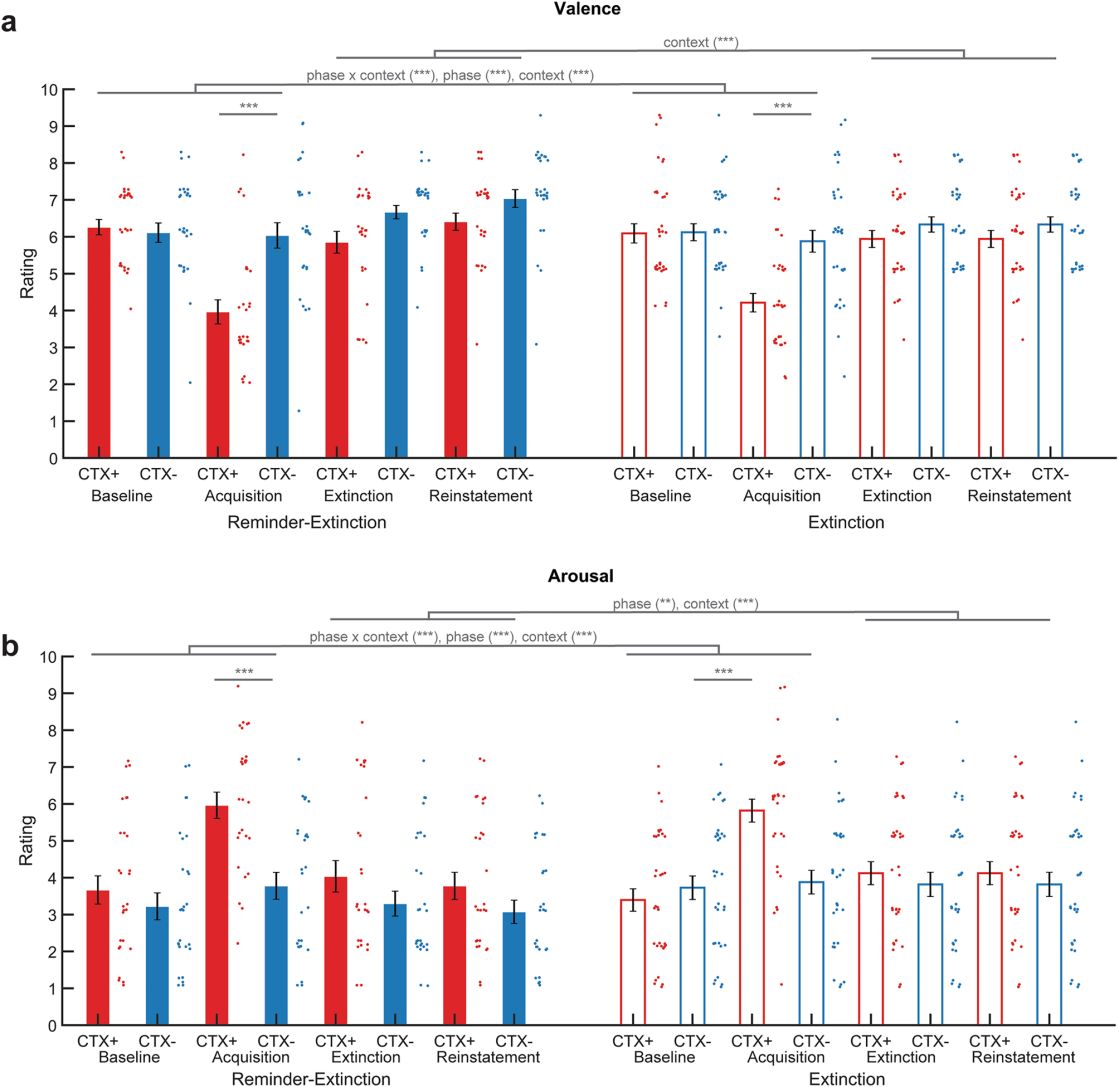
An isolated reminder before extinction did not influence retention of valence and arousal ratings. Context conditioning resulted in acquisition of subjective threat, which was subsequently extinguished, and re-extinguished after the reinstatement test under extinction conditions. Bar plots reflecting mean valence and arousal ratings before acquisition, after acquisition, after extinction and after reinstatement of context conditioning for the threat (CTX+, red) and safe context (CTX−, blue) in the Reminder-Extinction (solid bars) and Extinction (open bars) groups. Context conditioning resulted in (**a**) lower valence ratings and (**b**) higher arousal ratings. Error bars = s.e.m., adjacent dots represent jittered individual data-points. **p < 0.01, ***p < 0.001.

Valence and arousal ratings were obtained before and after the contextual threat conditioning task, after extinction, and after the reinstatement test. The valence and arousal ratings showed successful acquisition of differential context conditioned threat memories, and this effect decreased but persisted after extinction and reinstatement (Fig. 3). Yet we found no evidence for an effect of the reminder-extinction procedure on these subjective measures of contextual threat conditioned memory.

Valence ratings and arousal ratings were subjected to a phase (baseline, after acquisition) × context (CTX+, CTX−) × group (R-Ext, Ext) rmANOVA to explore whether both groups showed similar acquisition of contextual threat conditioned memories. For valence ratings, this revealed an interaction of phase and context (F_1,58_ = 35.790, p < 0.001, η^2^ = 0.382), and a significant main effect of phase (F_1,58_ = 52.368, p < 0.001, η^2^ = 0.474) and context (F_1,58_ = 33.563, p < 0.001, η^2^ = 0.367). For arousal ratings, we found an interaction of phase and context (F_1,58_ = 46.287, p < 0.001, η^2^ = 0.444), and main effects of phase (F_1,58_ = 40.566, p < 0.001, η^2^ = 0.412) and context (F_1,58_ = 36.430, p < 0.001, η^2^ = 0.386). Neither valence nor arousal ratings showed effects of group (all P’s > 0.15). Differential ratings (CTX+ − CTX−) increased after acquisition for both valence (t(59) = 5.829, p < 0.001, baseline: 0.05 ± 0.14, after acquisition; 1.9 ± 0.28) and arousal (t(59) = 6.939, p < 0.001, baseline: 0.02 ± 0.16, after acquisition: 2.1 ± 0.28). Baseline ratings were similar for the CTX+ and CTX− for valence (p > 0.7, CTX+: 6.17 ± 0.17, CTX−: 6.12 ± 0.17) and arousal (p > 0.9, CTX+: 3.5 ± 0.24, CTX−: 3.5 ± 0.24), while after the acquisition task, valence ratings lower for the CTX+ than the CTX− (t(59) = − 6.513, p < 0.001, CTX+: 4.1 ± 0.2), CTX−: 6.0 ± 0.22) and arousal ratings were higher for the CTX+ than the CTX− (t(59) = 7.252, p < 0.001, CTX+: 5.9 ± 0.23, CTX−: 3.8 ± 0.24). Valence ratings for the CTX+ decreased after acquisition (t(59) = − 9.791, p < 0.001, baseline: 6.17 ± 0.17, after acquisition 4.10 ± 0.20), while valence ratings for the CTX− did not change (p = 0.48). Arousal ratings for the CTX− did not change from baseline to after acquisition (p > 0.16), but arousal ratings for the CTX+ increased after acquisition (t(59) = 8.667, p < 0.001, baseline: 3.5 ± 0.24, after acquisition: 5.9 ± 0.23). Thus, both groups show a similar acquisition of a differential conditioned threat response in valence and arousal ratings.

To test whether the reminder-extinction procedure affected the recovery of valence and arousal ratings after completion of the reinstatement test, valence and arousal ratings were subjected to a time (after extinction, after reinstatement) × context (CTX+, CTX−) × group (R-Ext, Ext) rmANOVA. For valence, there was a main effect of context (F_1,58_ = 18.076, p < 0.001, η^2^ = 0.238) and phase (F_1,58_ = 4.825 p = 0.032, η^2^ = 0.077), but no interactions with group (all P’s > 0.07). Similarly, for arousal we found main effects of phase (F_1,58_ = 4.793, p = 0.033, η^2^ = 0.076) and of context (F_1,58_ = 12.071, p = 0.001, η^2^ = 0.172), but no interactions with group (all P’s > 0.2). Follow up *t* tests of mean ratings after the extinction and reinstatement sessions showed that valence ratings remained lower for the CTX+ than the CTX− (t(59) = − 4.048, p < 0.001, CTX+: 6.1 ± 0.16, CTX−: 6.6 ± 0.14) and arousal ratings remained higher for the CTX+ than the CTX− (t(59) = 3.345, p < 0.001, CTX+: 3.9 ± 0.23, CTX−: 3.4 ± 0.21). Differential ratings (CTX+ − CTX−) did not change from after extinction to after the reinstatement test (all Ps > 0.3). As our reinstatement test was carried out under extinction conditions, and the arousal ratings were taken after the end of this task, it is not surprising that we do not see any effect of reinstatement on differential arousal ratings measured after the reinstatement test.

### Retrospective shock estimation

**Figure 4:**
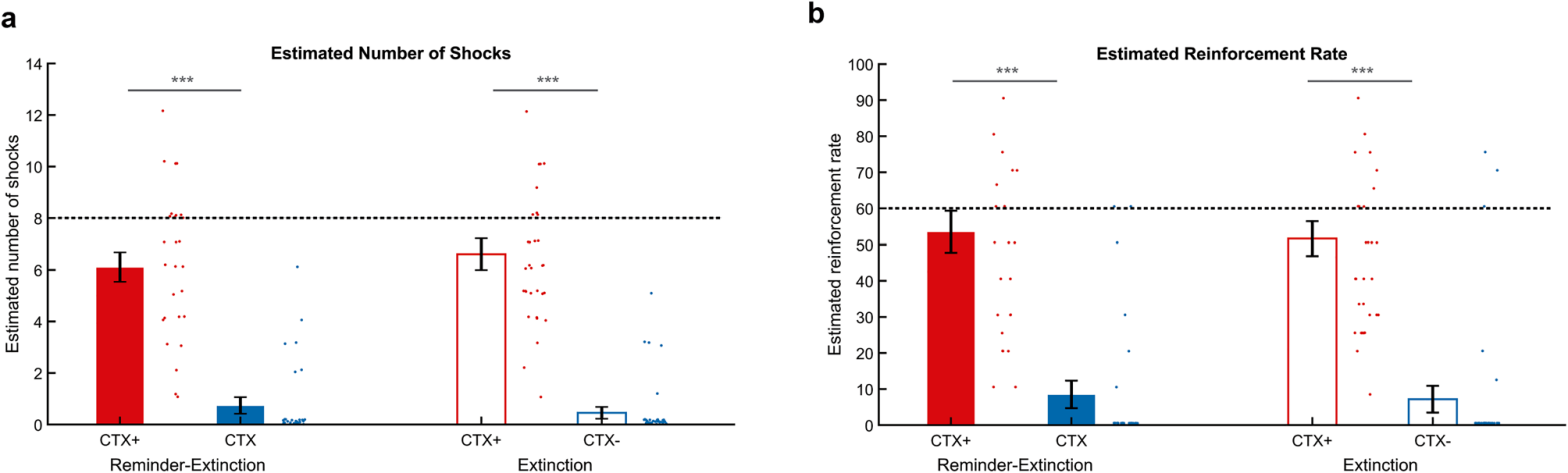
An isolated reminder before extinction did not affect explicit threat memory after context conditioning. Bar plots reflecting (**a**) the mean estimated number of shocks received during the acquisition task and (**b**) the estimated reinforcement rate during the acquisition task for the threat (CTX+, red) and safe context (CTX−, blue) in the Reminder-Extinction (solid bars) and Extinction (open bars), tested at the end of the experiment. Dashed line indicates (**a**) the actual number of shocks (8 in CTX+ only) and the actual reinforcement rate (60% in CTX+ only). Error bars = s.e.m., adjacent dots represent jittered individual data-points.

To test the effect of a reminder on retrospective shock estimation and awareness at the end of the study, shock estimates and contingency awareness for the acquisition task of day 1 were subjected to a context (CTX+, CTX−) × group (R-Ext, Ext) rmANOVA. For the estimated reinforcement rate, there was a main effect of context (F_1,58_ = 120.363, p = 0.000, η^2^ = 0.638), but no effect of group (p = 0.711), nor interaction (p = 0.948) (Fig. 4a). As a Kolmogorov–Smirnov test indicated that estimates for the reinforcement rate of the CTX− did not follow a normal distribution (D(60) = 0.473, p < 0.001), we deviated from the pre-registered test and used a Wilcoxon signed-rank test as non-parametric alternative to the paired *t* test. A follow-up Wilcoxon signed-rank test revealed that across both groups, the estimated reinforcement rate was higher for the CTX+ than the CTX− (Z = − 6.241, p < 0.001, CTX+: 52.5 ± 3.5%, CTX−: 7.8 ± 2.5%). For the number of shocks participants estimated to have received, there was also a main effect of context (F_1,58_ = 145.004, p < 0.001, η^2^ = 0.714), but no effect of group (p = 0.803) or interaction (p = 0.418) (Fig. 4b). As a Kolmogorov–Smirnov test also indicated that estimates for the reinforcement rate of the CTX− did not follow a normal distribution (D(60) = 0.482, p < 0.001), we again deviated from the pre-registered tests and followed up with a Wilcoxon signed-rank test. In both groups, the estimated number of shocks was higher for the CTX+ than the CTX− (Z = − 6.540, p < 0.001, CTX+: 6.4 ± 0.41, CTX−: 0.58 ± 0.18).

### Spatial memory test

To test how contextual threat conditioning and the reminder-extinction procedure would affect the participants’ ability to remember the spatial location of items in the contexts, we asked participants to indicate on a grid-map representation of the rooms where specific furniture items had been located.

**Figure 5:**
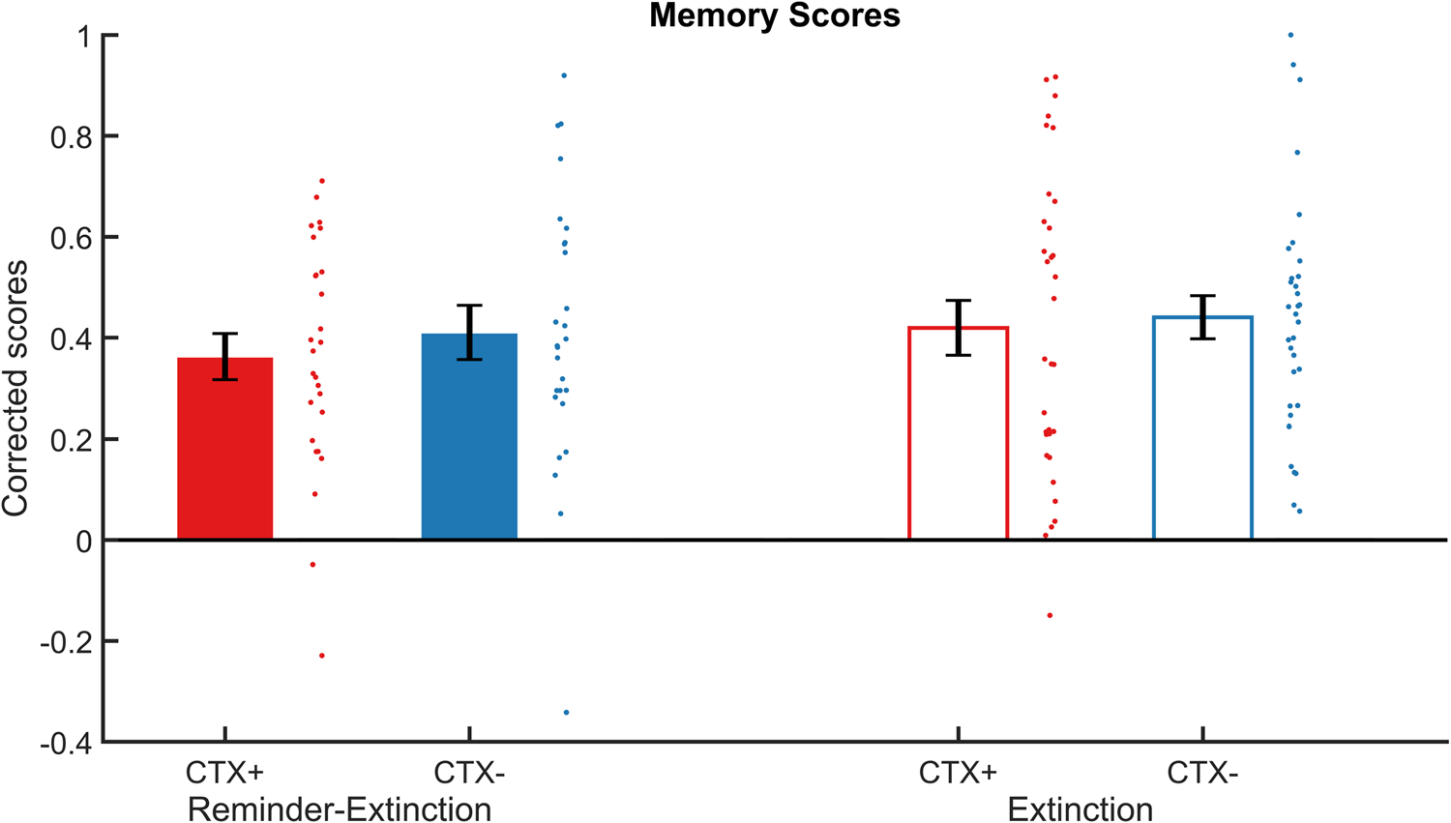
An isolated reminder before extinction did not affect memory of the location of items in each context. Bar plots reflect mean scores on the item location memory test for the threat (CTX+, red) and safe context (CTX−, blue) in the Reminder-Extinction (solid bars) and Extinction (open bars), tested at the end of the experiment. Participants remembered items from both contexts above chance level and there were no differences in location memory between contexts. A score of 0 indicates chance level. Error bars = s.e.m., adjacent dots represent jittered individual data-points.

Subjecting item-location memory scores (see Fig. 5) were subjected to a group (R-Ext, Ext) × context (CTX+, CTX−) rmANOVA revealed no interaction or main effects of group (all P’s > 0.5) and context (p > 0.17). To explore whether memory scores were above chance level, mean memory scores across groups and context were subject to a one-sample *t* test. Mean memory scores were above chance level of 0, a statistically significant difference of 0.41 (95% CI 0.34–0.47), t(59) = − 2.381, p < 0.001. Hence, participants remembered the location of the items in the rooms but contextual conditioning nor the reminder-extinction procedure affected memory.

### Avoidance

**Figure 6:**
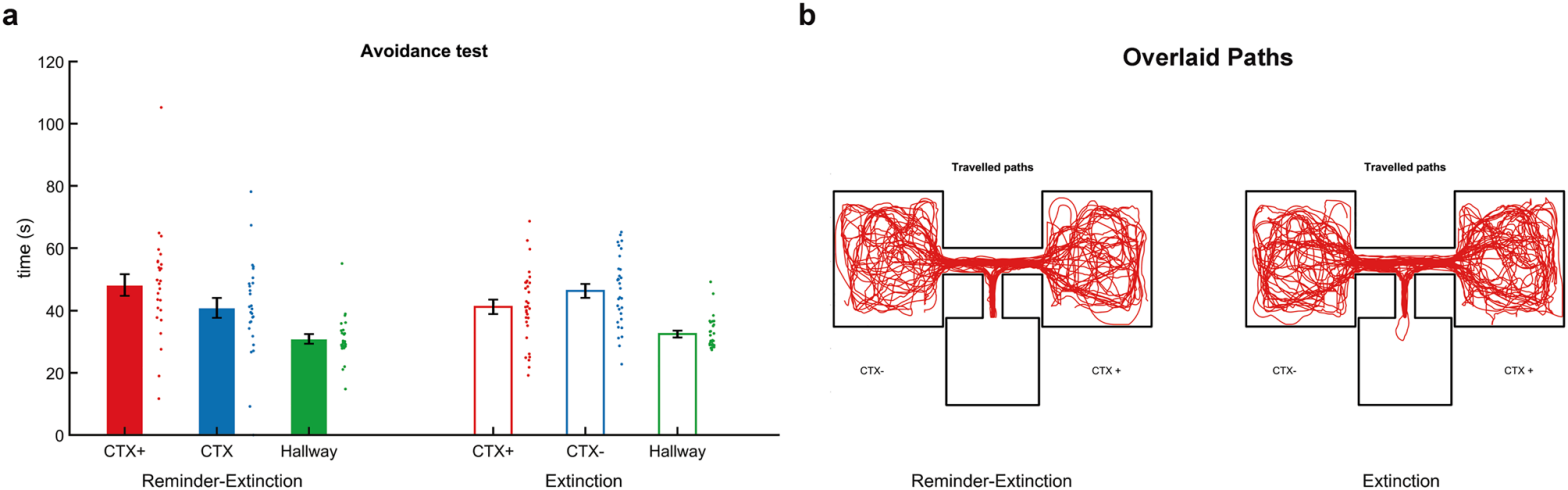
An isolated reminder before extinction did not affect free exploration behavior after re-extinction of contextual threat conditioned responses. (**a**) Bar plots reflect mean time spent in the threat (CTX+, red), safe context (CTX−, blue), and hallway (green) for the Reminder-Extinction (solid bars) and Extinction groups (open bars) tested at the end of the experiment. Participants spent similar amounts of time in the CTX+ and CTX−. Error bars = s.e.m., adjacent dots represent jittered individual data-points. (**b**) Travelled paths are similar in the Reminder-Extinction and Extinction group, and similar for the CTX+ and CTX−. Individual travelled paths are mirrored for a subset of participants to display the CTX+ on the left for all participants.

After the reinstatement test under extinction conditions and the spatial memory test, participants freely navigated through the contexts while performing a cover task that required exploration of the CTX+ and CTX−. To test whether return of contextual threat memory was associated with avoidance behaviour, the time spent in each context was compared. A group (R-Ext, Ext) × context (CTX+, CTX−) rmANOVA revealed no interaction or main effects of group or context, indicating that participants did not avoid the threat conditioned context (all P’s > 0.08) (Fig. 6). To further investigate whether a reminder before extinction might reduce avoidance of the threat conditioned context, we carried out a Chi-square test to check whether there was a difference between the first room that was entered (CTX+ or CTX−) between the two groups (R-Ext, Ext). In our pre-registration, we had planned to carry out a Fisher’s exact test, but given the fact that Fisher’s exact test is only used when at least one of the four cells of a 2 × 2 table contains less than five observations, and all of our cells had at least 12 observations, we decided a Chi-square test was more appropriate. The Chi-square test revealed no differences between groups in the first room that was visited (*Χ*^2^(2) ≥ 0.606, *p* = 0.604) (Table 1). Thus, participants explored both rooms equally and neither contextual conditioning nor the reminder-extinction procedure affected exploration behaviour.

**Table 1:** An isolated reminder before extinction did not affect the likelihood of avoiding the CTX+ on first entry.

	First room visited		Total
	CTX−	CTX+	
**Reminder-extinction**			
Count	12	15	27
Expected count	13.3	13.5	27
**Extinction**			
Count	18	15	33
Expected count	16.5	16.5	33
**Total**			
Count	30	30	60
Expected count	30	30	60

Crosstabulation of the first room visited for the Reminder-Extinction and Extinction group during free exploration at the end of the experiment.

### Skin conductance and heart-rate responses

As secondary measures of physiological responses we also obtained skin conductance and heart-rate responses. However, as these showed weak contextual threat conditioned responses at best we were unable to assess the influence of the reminder-extinction procedure on these measures. For completeness we have included the results of these measures in the Supplementary Information.

### Deviations of pre-registered design

In our pre-registered design, we planned to include an additional control group to test whether the effect of a reminder on the return of contextual threat conditioned memory was time-dependent. As the reminder-extinction procedure is thought to modify memory through a reconsolidation-update mechanism, we wanted to include an immediate memory test to gather evidence that the effect of a reminder was due to an interference with a reconsolidation process rather than immediate learning processes. This group would have been subjected to the spontaneous recovery and reinstatement tests, and all other tests planned for day 3, immediately after extinction on day 2. However, as we did not find an effect of the reminder-extinction procedure on the return of contextual threat conditioned memory, we did not test this additional control group. In addition, we planned to include 24 complete cases per group. To obtain 24 complete cases per groups, we tested thirty participants per group, after exclusion of participants that did not show evidence of differential conditioned threat responses according to pre-registered criteria, only 21 participants remained in the reminder-extinction group and 19 participants in the extinction group for critical results on FPS responses.

## Discussion

We investigated the efficacy of the reminder-extinction procedure to prevent the return of contextual threat conditioned memory in humans. On day 1, participants in both the reminder-extinction and extinction group acquired comparable discriminatory contextual threat conditioned FPS responses. Both groups exhibited initial retention during extinction on day 2 and full extinction over the course of the task. In contrast to our hypothesis, both the reminder-extinction and the extinction group showed comparable spontaneous recovery and reinstatement of FPS responses. We also found no effects of the reminder-extinction procedure on context conditioned valence and arousal ratings or explicit memory for the received shocks. Thus, we found no evidence that the reminder-extinction procedure is a more effective procedure to modify contextual threat conditioned memories in humans as compared to regular extinction.

There are several potential explanations as to why we found no effect of an isolated reminder before extinction on the return of threat responses. In line with a previous studies that observed no effect of the reminder-extinction procedure on the prevention of category conditioned threat responses^36^ and no effect on cues presented within a contextual frame^71^, the current contextual conditioning paradigm may place greater demands on hippocampal memory mechanisms than cue-conditioning, rendering the threat memory less sensitive to attenuation by the reminder-extinction procedure. This could suggest that threat conditioned hippocampal-dependent memories might be less sensitive or even insensitive to attenuation through the reminder-extinction procedure^8,94,95^. In support of this hypothesis, it has been suggested that the use of expectancy ratings negatively modulates the effect of the reminder-extinction procedure on the return of fear in humans, potentially by increasing declarative awareness of contingencies and thereby increasing hippocampal-dependence^96^. However, this explanation stands in contrast to previous studies in rodents that shown enhanced efficacy of the reminder-extinction procedure as compared to regular extinction for the attenuation of contextual fear memories in mice^65^ and rats^27^.

Since the first report on the efficacy of the reminder-extinction procedure to persistently attenuate fear in humans^46^, numerous studies have provided evidence for the enhanced efficacy of the reminder-extinction procedure as compared to regular extinction^47,50–54,67,97–102^. At the same time, there is a growing body of literature that does not find enhanced efficacy of the reminder-extinction procedure, raising the possibility that the it is highly dependent on potential boundary conditions, may be subject individual variation, or otherwise difficult to replicate (for reviews, see Refs.^103–105^). We suggest that our findings are in line with the literature that is unable to replicate the reminder-extinction effects on conditioned threat memories in humans^36,55,56,58–62,71^, where we note that ours diverges specifically in that we investigated contextual threat memories using iVR. The limited replicability may not be limited the reminder-extinction procedure, but could generalize to reconsolidation-based interventions^106,107^. It would therefore be worthwhile to explore if other interventions such as beta-blockers^18,108^, propofol^33^, electrical brain stimulation^35^, or other behavioural interventions^109^ are capable of permanently attenuating contextual threat memories in humans. By doing so, we will hopefully reach a more mechanistic understanding of how post-retrieval interventions can impact memories.

A recent meta-analysis by Kredlow et al. (2016) has identified several potential boundary conditions of postretrieval extinction effects for threat memories in humans, including the number of acquisition trials, shock duration and the fear-relevance of the CS. Specifically, it was suggested that in two previous human studies, a short shock duration of 2 ms might have contributed to negative or null findings and it should be noted that the current study also used this short shock duration^55,60^. In addition, although our individual trials were of long duration compared to cue-conditioning studies, the relatively low number of trials used may also have negatively impacted our findings^96^. On the other hand, the use of fear-irrelevant stimuli, as opposed to fear-relevant stimuli, has been identified as a positive modulator, and would be expected increase the efficacy of the reminder-extinction procedure in the current study^96^. In a previous study investigating contextual conditioning using framed fear-relevant pictures, it was suggested that the use of fear-relevant stimuli may have contributed to the negative finding^71^. Contrasting this finding to our present negative finding using fear-irrelevant stimuli suggests that potential explanations of (non-) replication in terms of such boundary conditions is interesting but also complex and potentially non-definitive*.*

More interestingly, instead of post-hoc identification, a number of studies have experimentally investigated the influence of potential boundary conditions and inter-individual differences on the efficacy of the reminder-extinction procedure. In humans, such studies have suggested that factors such as reminder duration^53^, reminder stimulus type^67,110–112^, threat-response measure avoidance behaviours^113^, inclusion of on-line expectancy ratings^114^ and genetics^115^ may influence the effectiveness of the reminder-extinction procedure. On the other hand, the reminder-extinction procedure has been shown to be equally effective for young and old memories^52^ and for fear-relevant and fear-irrelevant stimulus material^98^. It has previously been suggested that the use of startle probes may influence SCRs^55^, and it could be interesting for future studies to prospectively test in a between-subject design whether FSP and SCR are differentially sensitive to reminder-extinction interventions.

Alternatively, even though we observe reactivation of contextual threat memory as indexed by threat-potentiated startle that, critically, did not trigger extinction learning, our reminder procedure may have failed to reactivate memory in such a way that it resulted in destabilization of the memory. If indeed the efficacy of the reminder-extinction procedure depends memory destabilization and disruption of a reconsolidation process, generation of a prediction error during the reminder may be critical for successful destabilization^116,117^ (for a review, see^118,119^). It would be of interest for future prospective studies to investigate the conditions that result in the destabilization of contextual threat memories in humans. Another explanation is that because we observed an unexpected difference between groups at the start of acquisition, the reminder-extinction group may have conditioned more strongly and the lack of a difference between groups could potentially reflect a diminishment of threat recovery in the reminder-extinction group after all. However, such an explanation does not fit with our a priori hypotheses. Moreover, considering that we randomly assigned participants to either group it is surprising to observe group differences during the initial phase of acquisition. This group difference was driven by a difference in responses in the CTX− room, not CTX+, during the early phase of acquisition. At the end of acquisition and at the start of extinction both groups show comparable differential contextual threat conditioned responses. To us this suggests that both groups acquired, consolidated, and retained comparable differential contextual threat conditioned responses, rendering this alternative explanation unlikely.

A potential limitation of the current study is that both groups underwent an equal number of visits to the conditioned context during extinction training, and the reminder visit, also carried out under extinction conditions, thus constitutes additional exposure in the R-Ext group. However, we found that the final trials of extinction training seem to have a negligible (i.e. non-significant) contribution to extinction learning. In addition, if the additional exposure to the CTX+ under extinction conditions would have had an effect, we would expect to find attenuated spontaneous recovery and reinstatement in the R-Ext group, which we did not observe. A further limitation may be that the sample size of the current study is similar to previous studies investigating the effect of the reminder-extinction procedure on cue-conditioning in humans^46,60^. For studies in humans, a meta-analysis by Kredlow et al. has reported a significant, small-to-moderate effect of the reminder-extinction procedure for further reducing the return of fear in humans as compared to standard extinction^96^. For future studies, using increased sample sizes would contribute to a more convincing (non-) replication of the original findings^120^.

For the translation from laboratory research on reconsolidation to clinical applications, it is relevant to keep in mind that symptoms in stress- and anxiety-related disorders are not limited to maladaptive threat responses but also include subjective feelings, episodic memories, and avoidance behaviours. In the current study we found no evidence that conditioned arousal and valence ratings were diminished after the presentation of a brief reminder before extinction. We also probed the influence of the reminder-extinction procedure on episodic memory, and found that participants in both groups were equally able to retroactively estimate the number of shocks and the reinforcement rate they had experienced. We also did not find an effect of an isolated reminder before extinction on item-location spatial memory. These findings are in contrast to human and rodent studies which indicate that reconsolidation-based interventions can impair episodic^10,31,32,35,71,121^ and spatial memories^122,123^. They are also in contrast to previous studies in rodents and humans that suggest that a reminder in the absence of reconsolidation-interventions, or when interventions fail, can strengthen aversive episodic and spatial memories^35,36,124^. Yet, our findings may be in line with previous studies showing that reconsolidation-based interventions leave explicit knowledge about contingencies intact^18^. Hence, our results suggest that the reminder-extinction procedure fails to attenuate subjective feelings and episodic memories related to an aversive context.

At the start of the experiment, we explicitly instructed participants that their memory for item location would be tested. As a result, item-location memory may be strongly encoded and less sensitive to reconsolidation(-interventions) than incidentally encoded memories (^36^, but see Ref.^35^ for instructed memory test albeit with weak memory performance). In addition, we found no emotional enhancement effect^125,126^ of contextual threat conditioning on spatial item-location memory, akin to previous work on context conditioning in humans^40^. This might be because the location of individual items carries little predictive value in the contextual threat learning experience, which may require a conjunctive representation of the whole space^127^, highlighting the complicated interaction between anticipation, attention and arousal on memory^128^. Therefore, the lack of an effect of the reminder-extinction procedure on item-location memory may alternatively be explained by the suggestion that interventions targeting reconsolidation may only reduce the emotional enhancement of episodic memories^35,95^.

Given that avoidance behaviour can diminish before explicit threat expectancies have changed^129^, we also investigated whether a reminder before extinction could reduce avoidance of the threat-conditioned context by tracking participants’ free exploration of the contexts after spontaneous recovery and reinstatement of the conditioned threat response. We did not find evidence for avoidance of the threat-conditioned context, as participants in both the reminder-extinction and extinction group spent comparable amounts of time in both the threat-conditioned and the safe context and were equally likely to visit either context first. Given the lack of avoidance behaviour, we are unable to say whether a reminder before extinction could affect avoidance behaviour. However, as the avoidance test was conducted after spontaneous recovery and reinstatement tests that were carried out under extinction conditions, it may not be surprising that our test did not trigger avoidance. Regardless, we think such avoidance test is an interesting new tool for the emotional memory field when tested immediately after contextual threat conditioning. Especially in light of the recent finding that a beta-adrenergic reconsolidation-intervention allowed people with spider phobia to overcome avoidance behaviours, upon which their subjective feelings of threat also diminished^129^, highlighting the interaction between threat-related defensive responses, avoidance behaviours, and cognitive representations of fear^130^.

In conclusion, we did not find evidence for the prevention of the return of contextual threat memories using the reminder-extinction paradigm in humans. At present, it is unclear whether this could be because the reminder-extinction procedure is ineffective in modifying hippocampus-dependent contextual threat memories specifically, threat memories more generally, or because of alternative reasons. It would therefore be worthwhile to explore if other interventions such as beta-blockers^18,108^, propofol^33^, electrical brain stimulation^35^, or other behavioural interventions^109^ are capable of permanently attenuating contextual threat memories in humans. Alternatively, even though we observed reactivation of contextual threat memory as indexed by threat-potentiated startle, our reminder procedure may simply have failed to reactivate memory in such a way that it resulted in destabilization of the memory. Many explanations and boundary conditions to the (non-)destabilization of memory can be proposed post-hoc, yet the exact conditions that allow the reactivation and destabilization of memories are rarely experimentally and prospectively investigated^121,131,132^, particularly in humans. We therefore strongly encourage future studies to investigate the mechanisms underlying the destabilization of memories. Regardless of such future investigations, our current results raise the possibility that the reminder-extinction procedure does not lead to a persistent attenuation of contextual conditioned threat memories in humans.

## Supplementary information

Supplementary Information.

## Data Availability

The datasets generated during and/or analysed during the current study are available from the corresponding author on request.
