# Supplementary material for: Investigating the efficacy of the reminder-extinction procedure to disrupt contextual threat memories in humans using immersive Virtual Reality
Source: Sci Rep. 2020 Oct 12;10:16991. doi: 10.1038/s41598-020-73139-4 (PMC7550330; doi:10.1038/s41598-020-73139-4)
Supplement: Supplementary file 1 — Supplementary Information. [file 41598_2020_73139_MOESM1_ESM.pdf]

Investigating the efficacy of the reminder-extinction procedure to disrupt contextual threat memories in humans using immersive Virtual Reality

Maxime C. Houtekamer, Marloes J.A.G. Henckens, Wayne E. Mackey, Joseph E. Dunsmoor, Judith R. Homberg, Marijn C.W. Kroes

Supplementary information

Acquisition and extinction of fear potentiated startle responses

On day one, we observed comparable acquisition of discriminatory contextual threat conditioned startle responses between both groups (Figure 2a). A group (R-Ext, Ext) x phase (early, late) x context (CTX+, CTX-) rmANOVA revealed an interaction effect of group x context ($F_{1,38}$=5.037, p=0.031, η2=0.117), a main effect of phase ($F_{1,38}$=81.418, p<0.001, η2 = 0.682), and a main effect of context ($F_{1,38}$=87.074, p<0.001, η2=0.696), with no other main effects or interactions. A follow-up independent t-test on the difference between startle responses in the CTX+ versus CTX- revealed unexpected greater differential responses in the R-Ext than Ext group (t(38)=2.244, p=0.031, R-Ext: 5.0±0.70, Ext: 3.0±0.50). Another follow-up independent samples t-test revealed no group differences in FPS responses in the CTX+ (t(38)=1.082, p=0.286, R-Ext: 53.2±0.42, Ext: 52.5±0.44) but a difference in startle responses in the CTX- at trend (t(38)=-1.998, p=0.053, R-Ext: 48.2±0.51, Ext: 49.5±0.38). Across both groups we observed greater startle responses across both contexts in the early than late phase (t(39)=8.692, p<0.001, early: 54.1±0.41, late: 47.5±0.46), consistent with the normal habituation of startle responses over time. Importantly, across both groups we observed greater startle in the CTX+ than CTX- (t(39)=9.001, p<0.001, CTX+: 52.9±0.30, CTX-: 48.8 ± 0.34), indicating that both groups acquired differential contextual threat conditioned responses. Yet, as we observed an unexpected group x context effect we decided to explore this potential group difference further and tested FPS seperately for the early and late phase of acquisition, and compared startle responses in the CTX+ and CTX- with responses in the hallway as a control condition. A group (R-Ext, Ext) x context (CTX+, CTX-) rmANOVA for the early phase of acquisition revealed a trend-level interaction effect of group x context ($F_{1,38}$=53.311, p=0.077, η2=0.080) and a main effect of context ($F_{1,38}$=53.787, p<0.001, η2 = 0.586). A group (R-Ext, Ext) x context (CTX+, CTX-) rmANOVA for the late phase of acquisition only revealed a main effect of context ($F_{1,38}$=30.467, p<0.001, η2=0.445) and no trend for group x context interactions ($F_{1,38}$=1.643, p=0.208, η2=0.041). Thus, critically, in the late phase of acquisition, both groups showed comparible differences between startle responses in the CTX+ and CTX- indicating comparable acquisition of contextual conditioned threat responses.

On day two, both groups underwent succesful extinction of contextual threat conditioned FPS, which was preceded by an isolated reminder for the R-Ext group. During the reminder, we observed greater startle responses in the CTX+ than hallway (t(20)=3.114, p=0.005, startle responses of 61.8 ± 2.8 in the CTX+ and 49.26 ± 1.8 in the hallway, as participants did not traverse the CTX- during the reminder, a comparison between FSP in the CTX+ and CTX- was not possible). Thus, the reminder resulted in reactivation of the contextual threat conditioned memory in the R-Ext group. During the extinction task, both groups exhibited comparable extinction of FPS responses (Figure 2a). A group (R-Ext, Ext) x phase (early, late) x context (CTX+, CTX-) rmANOVA on FPS responses during the extinction task revealed an interaction of phase x context ($F_{1,37}$=8.552, p=0.006, η2=0.188) and a main effect of phase ($F_{1,37}$=217.726, p<0.001, η2=0.855), with no other main effects or interactions. Although there was a main effect of context at trend (p=0.058), there were no main or interaction effects of group (All p's>0.16). Follow-up paired t-tests revealed greater differential FPS responses in the early phase compared to the late phase (t(38)=2.787, p=0.008, early: 2.58±1.0, late: 0.18±0.46). Specifically, FPS responses in the CTX+ were greater than in the CTX- in the early phase (t(39)=2.185, p=0.035, CTX+: 55.5±0.80, CTX-: 53.3±0.54) but not the late phase (t(39)=-0.393, p=0.696, CTX+: 45.6±0.32, CTX-: 45.8±0.37), indicating that both groups intially exhibited retention of contextual threat conditioned FPS responses that fully extinguished over the course of the extinction task.

As the R-Ext group was reminded of the CTX+ by a single exposure under extinction conditionings, and both the R-Ext and the Ext group both underwent extinction training of 10 visits to each context under extinction conditions, we aditionally explored whether any additional learning seems to take place in the final extinction trial, by comparing FPS during the final two visits to the CTX+. A group (R-Ext, Ext) x Trial (9, 10) x Startle probe (1, 2) rmANOVA did not reveal any main or interaction effects of trial, probe or group (All p's>0.1), suggesting that contribution of the final trial to extinction learning is negligble.

On day three, spontaneous recovery of FPS was tested under extinction conditions. To examine whether the lack of differential startle responses in the early phase could be due to a generalization of the startle potentiation to the CTX-, we further explored these effects by comparing responses in the CTX+ and CTX- with responses in the hallway for each phase. These unplanned comparisons revealed greater responses in the CTX+ and CTX- compared to the hallway in the early phase of spontaneous recovery (t(39)=2.481, p=0.018 for comparison between the CTX+ and hallway, CTX+: 54.1±1.3, hallway: 50.1±0.80, and t(39)=2.765, p=0.009 for comparison of the CTX- and hallway, CTX+: 54.2±1.1), but no difference between the CTX+ and hallway or CTX- and hallway in the late phase of spontaneous recovery (all Ps > 0.1).

Scoring for the Spatial Memory Task

Participants placed images of furniture items that had been present in the rooms on a spatial grid representation of each room (see Figure S1A-C). According to our pre-registration, we planned to calculate the percentage of correct answers for items in the CTX+ and CTX-. In order to differentiate between answers that were close but did not indicate the exact location of items, and answers that were wrong, we applied a graded scoring, where exactly correct answers were worth 1 point, close answers 0.5 points, remotely correct answers 0.2 points and completely incorrect answers were worth 0.1 points (see Figure S1D).

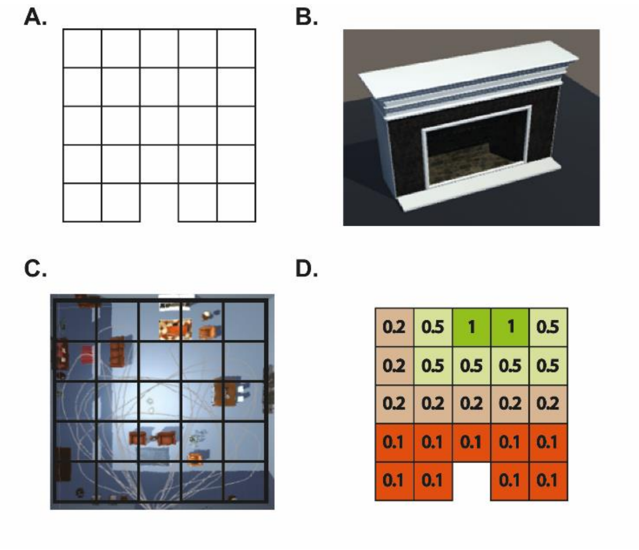

Figure S1. Impression of the spatial memory task. Participants were presented with an answer grid (A.) for every question and room separately. They were presented with pictures of objects that were located in the CTX+ and CTX- (B., for example), and asked to indicate the location of this object in each room on the answer grid. Correct locations were counted as any grid squares that contained a part of this object, as represented in C. A graded scoring system was applied (D.) where the exact correct location was worth 1 point and neighbouring grid locations were worth 0.5 points. Grid locations located two cells away from the correct location were worth 0.2 points and all other cells were worth 0.1 points.

As displayed in Figure S2, a Monte-Carlo simulation (1000 simulations) showed that the mean score based on chance level differs for the two different contexts for some of the questions. To correct for these differences, we performed a correction for chance level. Scores for each question and context for each participant were corrected by subtracting the chance-level mean and subsequently dividing by the maximum score minus the chance-level mean.

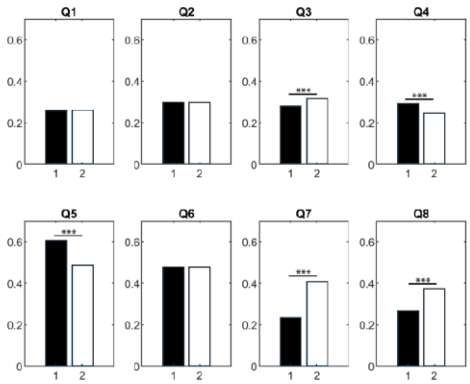

Figure S2. Mean scores for context one (black, 1) and context 2 (white, 2) for all 8 items of the spatial memory questionnaire resulting from a Monte-Carlo simulation. For questions 3, 4, 5, 7 and 8, the mean score at chance level is different for the two different contexts. Triple asterisk denote significance at the p<0.001 level.

Skin conductance responses

In our pre-registration, we specified that we would only include participants who showed successful conditioning during the acquisition phases, measured by a greater startle response in the threatening compared to the safe context. However, as non-responders on FPS are not necessarily the same as non-responders on skin conductance response (SCR) measures, we included for SCR analysis participants that showed a greater SCR in the threatening compared to the safe context. As we measured SCR both to the startle probe and to transitions into the contexts, we in- and excluded participants separately for the two SCR measures.

SCR in response to startle probes

For SCRs to startle probes, fourteen (out of twenty-seven) participants from the R-Ext and sixteen (out of thirty-three) participants from the Ext group showed differential SCRs during acquisition and were included in the analyses (see Figure S3). To explore whether there were group-differences over the course of acquisition, we carried out a phase (early, late acquisition) x context (CTX+, CTX-) x group (R-Ext, Ext) rmANOVA. This revealed a main effect of phase ($F_{1,29}$=5.997, p=0.021, η2=0.171) and of context ($F_{1,29}$=25.579, p<0.001, η2=0.469). Follow-up t-tests revealed that SCRs decreased from the early to the late phase (t(30)=2.472, p=0.019, early: 1.77±0.22, late: 1.36±0.18), and across the acquisition phase, SCRs in the CTX+ were larger than in the CTX- (t(30)=4.679, p<0.001, CTX+: 1.82±0.21, CTX-; 1.31±0.18). Both groups also showed comparable levels of extinction. A phase (early, late extinction) x context (CTX+, CTX-) x group (R-Ext, Ext) rmANOVA revealed an interaction effect of phase x context ($F_{1,29}$=3.962, p=0.024, η2=0.146) and a main effect of phase ($F_{1,29}$=14.278, p=0.001, η2=0.330). Follow-up t-tests revealed lower

differential SCRs in the late phase of extinction as compared to the early phase (t(30)=2.336, p=0.026, early: 0.33±0.13, late: -0.07±0.10). During the early phase, SCRs to startle probes in the CTX+ were greater than in the CTX- (t(30)=2.588, p=0.015, CTX+: 2.38±0.27, CTX-: 2.04±0.24) but in the late phase there was no longer any difference (p=0.481, CTX+: 1.55±0.16, CTX-: 1.63±0.18), demonstrating that there was successful extinction of the contextual threat conditioned SCR.

A reminder did not prevent spontaneous recovery of the conditioned threat response. To test the effect of a reminder on spontaneous recovery of threat responses, SCRs were subjected to a phase (early, late recovery test) x context (CTX+, CTX-) x group (R-Ext, Ext) rmANOVA. There were no interaction effects with or main effect of group (all Ps > 0.17), only a main effect of phase ($F_{1,29}$=16.790, p<0.001, η2=0.367) and a main effect of context at trend ($F_{1,29}$=2.904, p=0.99, η2=0.087). Follow-up t-tests showed differential responses did not change, while SCRs to startle probes dropped in both the CTX- (t(30)=4.156, p<0.001, early: 2.24±0.25, late: 1.73±0.20) and the CTX+ (p=0.030, early: 2.28±0.25, late: 1.95±0.25. This demonstrates retention of the contextual threat conditioned SCR, as re-extinction to the CTX- occurred more rapidly than to the CTX+.

In the transition from late extinction to early spontaneous recovery, we see a generalized increase of SCRs, with no evidence for an effect of the reminder-extinction procedure. To test for the increase in SCRs, responses were subjected to phase (late extinction, early recovery test) x context (CTX+, CTX-) x group (R-Ext, Ext) rmANOVA. There were no main or interaction effects of group (all Ps > 0.09), only a main effect of phase ($F_{1,29}$=8.246, p=0.008, η2=0.221). SCRs in both the CTX+ and CTX- were greater during early spontaneous recovery than during late extinction (t(30)=2.631, p=0.013, late extinction: 1.59±0.16, early spontaneous recovery: 2.26±0.25), which is consistent with a general increase in arousal at the start of a new experimental session.

The reinstatement test shows evidence for contextual threat conditioned SCRs in the CTX+ but does not reveal any effect of the reminder-extinction procedure. To test the effect of a reminder-extinction on reinstatement of threat responses, SCRs were subjected to phase (early, late reinstatement test) x context (CTX+, CTX-) x group (R-Ext, Ext) rmANOVA. There were no main or interaction effects of group (all Ps > 0.08), only a main effect of phase ($F_{1,29}$=18.710, p=0<0.001, η2=0.392). Follow-up t-tests showed differential responses did not change, while SCRs to startle probes dropped in both the CTX- (t(30)=2.600, p=0.014, early: 1.80±0.18, late: 1.39±0.14) and the CTX+ (t(30)= 2.355, p=0.023, early: 1.92±0.23, late: 1.53±0.16). Consistent with the pattern observed during spontaneous recovery, slower re-extinction to the CTzX+ suggests that the contextual threat conditioned SCRs are retained.

To test for the increase in responses, SCRs were subjected to phase (late recovery test, early reinstatement test) x context (CTX+, CTX-) x group (R-Ext, Ext) rmANOVA. There were no effects (all Ps > 0.067). As reinstated responses often extinguish rapidly, we also submitted reinstatement index scores (first trial of reinstatement test - last trial of recovery test) to a context (CTX+, CTX-) x group (reminder vs. no reminder) 2 x 2 repeated measures ANOVAs. There were no effects (all Ps > 0.129).

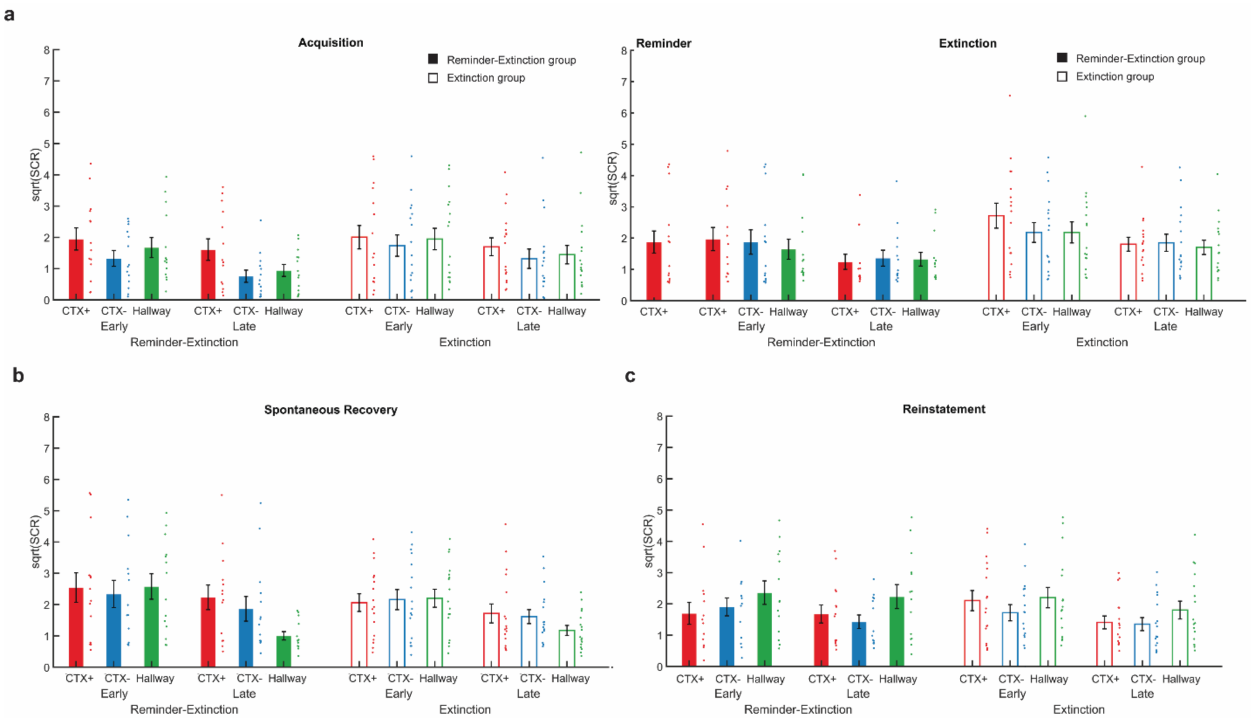

Figure S3. Skin conductance responses to startle probes

SCRs to transitions

For SCRs upon entry of the CTX+ and CTX-, fifteen (out of twenty-seven) participants from the R-Ext and eighteen (out of thirty-three) participants from the Ext group showed differential SCRs during acquisition and were included in the analyses (see Figure S4). Both groups show similar acquisition of contextual threat conditioned SCRs on day 1, as demonstrated by a phase (early, late acquisition) x context (CTX+, CTX-) x group (R-Ext, Ext) rmANOVA revealing only an interaction effect of phase x context ($F_{1,31}$=6.207, p=0.018, η2=0.167), a main effect of phase ($F_{1,31}$=20.043, p<0.001, η2=0.393) and a main effect of context ($F_{1,31}$=39.051, p<0.001, η2=0.557). Follow-up t-tests reveal a decrease in the differential SCR during acquisition (t(32)=2.525, p=0.017, early: 0.63±0.12, late: 0.21±0.09), while SCRs for transitions into the CTX+ remain greater than SCRs for transitions into the CTX- (t(32)=5.01, p<0.001 for the early phase, CTX+: 1.36±0.15, CTX-: 0.73±0.11, and t(32)=2.298, p=0.028 for the late phase, CTX+: 0.71±0.10, CTX-:

0.50±0.08). At the start of extinction, there was little evidence for retention for contextual conditioned SCRs to transitions into the CTX+. A phase (early, late extinction) x context (CTX+, CTX-) x group (R-Ext, Ext) 2x2x2 rmANOVA only revealed a main effect of phase at trend ($F_{1,31}$=3.963, p=0.055, η2=0.113). An exploratory t-test for differences in SCRs for transitions into the CTX+ and CTX- during the early phase of extinction revealed no difference. This suggests that as a measure of contextual threat conditioned memory, SCRs to transitions into the different contexts may be limited. This could be because participants never receive any shocks during the first five seconds in each context, as over the course of learning the latency of the SCRs shifts towards the moment at which shocks are anticipated (e.g. Prenoveau, Craske, Liao, & Ornitz, 2013). Although we carried out the pre-registered tests to investigate spontaneous recovery and reinstatement of SCRs, these only revealed an effect of phase, and additional t-tests did not reveal any differential responses for CTX+ and CTX- and did not reveal any changes in differential responses.

To test the effect of a reminder on spontaneous recovery of threat responses, SCRs were subjected to phase (early, late recovery test) x context (CTX+, CTX-) x group (R-Ext, Ext) rmANOVA. There were no main interaction effects of group (all Ps > 0.2), only a main effect of phase ($F_{1,31}$=6.736, p=0.014, η2=0.178). A follow-up t-test revealed a decrease in SCR responses during the spontaneous recovery test (t(32)=2.623, p=0.013, early: 1.53±0.23, late: 1.08±0.10).

To test for the increase in SCRs, responses were subjected to phase (late extinction, early recovery test) x context (CTX+, CTX-) x group (R-Ext, Ext) rmANOVA. There were no main or interaction effects of group (all Ps > 0.17), only a main effect of phase ($F_{1,31}$=4.919, p=0.034, η2=0.137). A follow-up t-test revealed an increase in SCR responses during early spontaneous recovery as compared to late extinction (t(32)=2.262, p=0.031, late spontaneous recovery: 1.04±0.08, early reinstatement: 1.53±0.23).

To test the effect of a reminder on reinstatement of fear responses, SCRs were subjected to a phase (early, late reinstatement test) x context (CTX+, CTX-) x group (R-Ext, Ext) rmANOVA. There were no main or interaction effects of group (all Ps > 0.2), only a main effect of phase ($F_{1,31}$=5.552, p=0.025, η2=0.152). A follow-up t-test revealed a decrease in SCR responses during the reinstatement test (t(32)=2.384, p=0.023, early reinstatement: 1.54±0.19, late reinstatement: 0.99±0.09).

To test for the increase in responses, SCRs were subjected to phase (late recovery test, early reinstatement test) x context (CTX+, CTX x group (R-Ext, Ext) rmANOVA. There were no main or interaction effects of group (all Ps > 0.2), only a main effect of phase ($F_{1,31}$=5.332, p=0.028, η2=0.147). A follow-up t-test

revealed an increase in SCR responses during the early reinstatement test as compared to the late phase of the spontaneous recovery test (t(32)=2.312, p=0.027, early: 1.08±0.10, late: 1.54±0.19).

As reinstated responses often extinguish rapidly, we also submitted reinstatement index scores (first trial of reinstatement test - last trial of recovery test) to a context (CTX+, CTX-) x group (R-Ext, Ext) 2x2 rmANOVA. There were no effects (all Ps > 0.13).

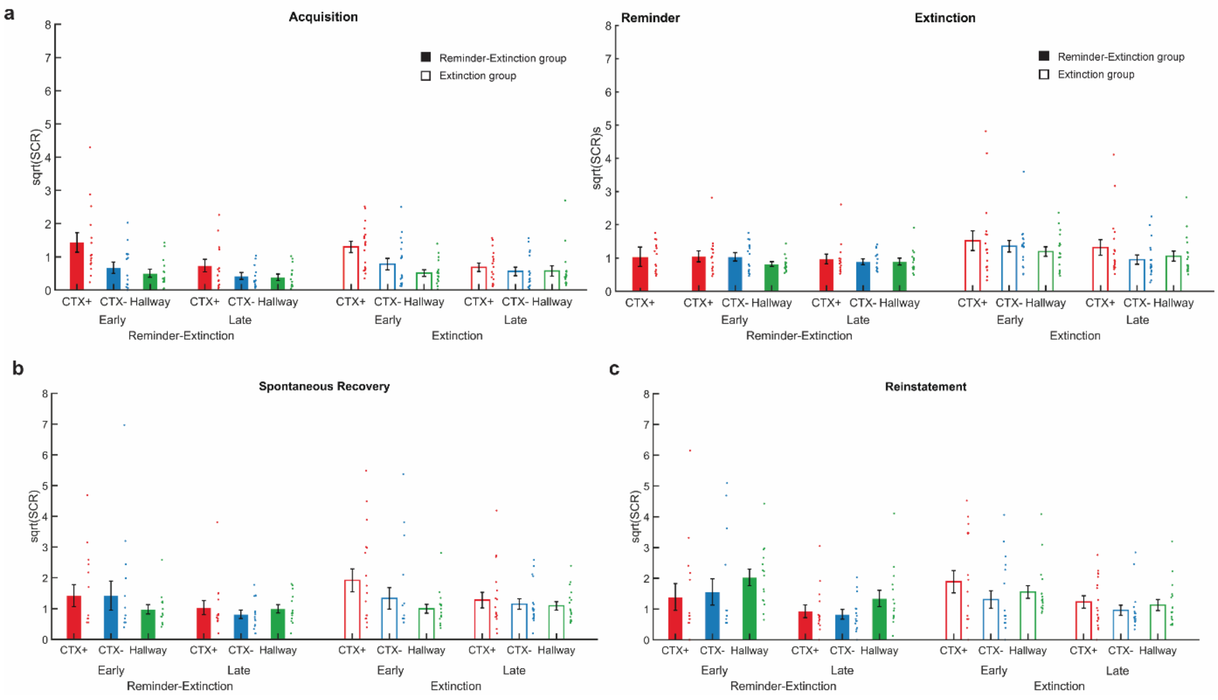

Figure S4. SCR in response to transitions

Heart rate changes during transitions

For both groups, heart rate shows a similar generalized conditioned threat response for both the CTX+ and CTX-. To explore whether HR responses showed evidence for conditioned threat responses to the CTX+, HR time courses were subjected to a timepoint x phase (early, late acquisition) x context (CTX+, CTX-) x group (R-Ext, Ext). This revealed a main effect of time point within the time course ($F_{2.642,124.184}$=21.325, p<0.001, η2=0.312). Follow-up t-test showed that relative to the baseline at 0s, HR at most time points (see table S1 below) showed a deceleration, but this deceleration is not specific to the CTX+.

Table S1. Significance levels for a comparison of each timepoint relative to baseline heart-rate during the early and late phase of the acquisition task. Results with significant comparisons (p<0.05) are shown in bold.

Timepoint	Early CTX+	Early CTX-	Late CTX+	Late CTX-

0.5s	t(48)=-1.911, p=0.062	t(48)=-0.114, p=0.910	t(48)=-1.523, p=0.134	t(48)=-1.333, p=0.189
1s	**t(48)=-2.145, p=0.037**	t(48)=-1.989, p=0.052	t(48)=-1.552, p=0.127	t(48)=-725, p=0.472
1.5s	**t(48)=-3.077, p=0.003**	t(48)=-1.947, p=0.057	t(48)=-1.777, p=0.082	**t(48)=-2.277, p=0.027**
2s	**t(48)=-4.074, p=0.000**	**t(48)=-2.652, p= 0.011**	**t(48)=-2.905, p=0.006**	**t(48)=-3.735, p=0.000**
2.5s	**t(48)=-3.791, p=0.000**	**t(48)=-3.806, p=0.000**	**t(48)=-3.078, p=0.003**	**t(48)=-5.131, p=0.000**
3s	**t(48)=-5.197, p=0.000**	**t(48)=-3.199, p=0.002**	**t(48)=-2.267, p=0.028**	**t(48)=-4.899, p=0.000**
3.5s	**t(48)=-5.182, p=0.000**	**t(48)=-3.150, p=0.003**	**t(48)=-3.028, p=0.004**	**t(48)=-3.362, p=0.002**
4s	**t(48)=-4.821, p=0.000**	**t(48)=-1.333, p=0.013**	**t(48)=-3.661, p=0.001**	**t(48)=-2.468, p=0.017**

At the beginning of extinction, HR in both groups shows a deceleration upon entry to the CTX+ and CTX-, but in the late phase, this is no longer the case (see table S2). To explore whether there was evidence for extinction of contextual threat conditioned HR responses to the CTX+, HR time courses were subjected to a phase (early, late extinction) x context (CTX+, CTX-) x group (R-Ext, Ext) rmANOVA. This revealed an interaction effect of phase x context x timepoint at trend ($F_{2.998,140.901}$=2.656, p=0.051, η2=0.053), an interaction effect of timepoint x group ($F_{2.693,126.593}$=4.101, p=0.010, η2=0.080), a main effect of phase ($F_{1,47}$=6.932, p=0.011, η2=0.129) and a main effect of timepoint ($F_{2.693,126.593}$=5.181, p=0.003, η2=0.099). Follow-up t-test showed that relative to the baseline at 0s, HR at most time points (see table S2 below) showed a deceleration during early extinction for both CTX+ and CTX- entries, while this was no longer the case during late extinction.

Table S2. Significance levels for a comparison of each timepoint to baseline during the early and late phase of extinction of the contextual threat conditioned HR response. Results from significant comparisons (p<0.05) are shown in bold.

Timepoint	Early CTX+	Early CTX-	Late CTX+	Late CTX-
0.5s	**t(48)=-3.168 , p=0.003**	t(48)=-.943 , p=0.350	t(48)=1.333, p=0.189	t(48)=-0.227, p=0.821
1s	**t(48)=-3.042 , p=0.004**	**t(48)=-2.425, p=0.019**	t(48)=-0,903, p=0.371	t(48)=-0.836, p=0.407
1.5s	**t(48)=-3.244 , p=0.002**	**t(48)=- 3.999, p=0.000**	t(48)=-1.030 , p=0.308	t(48)=-1.281, p=0.206
2s	**t(48)=-2.494 , p=0.016**	**t(48)=-4.095, p=0.000**	t(48)=-1.563, p=0.125	t(48)=-0.982, p=0.331
2.5s	**t(48)=-3.378 , p=0.001**	**t(48)=-4.531, p=0.000**	t(48)=-1.705, p=0.095	t(48)=-1.500, p=0.140
3s	**t(48)=-3.680 , p=0.001**	**t(48)=-4.249, p=0.000**	t(48)=-0.917, p=0.364	t(48)=-0.801 , p=0.427
3.5s	**t(48)=-3.089 , p=0.003**	**t(48)=-4.585, p=0.000**	t(48)=-1.523, p=0.134	t(48)=-1.852 , p=0.070
4s	**t(48)=-3.844 , p=0.000**	**t(48)=-4.661, p=0.000**	t(48)=-1.419 , p=0.162	t(48)=-2.380 , p=0.021

During the late phase of spontaneous recovery, we observed a stronger deceleration upon entry of the CTX+ as compared to the CTX-, but this deceleration was not affected by a reminder. To test the effect of a reminder on spontaneous recovery of fear responses, HR time courses were subjected to phase (early, late recovery test) x context (CTX+, CTX-) x group (R-Ext, Ext) 2x2x2 rmANOVA. There was an interaction effect of phase, context and time-point ($F_{2.645,121.689}$=3.333, p=0.027, η2=0.068). We ran follow-up paired t-tests comparing HR in the CTX+ and CTX- for each time points separately for the early phase and the late phase, and found stronger deceleration for the CTX+ in the late phase for the 2.0s time point, (t(47)=-2.077, p=0.043). This suggests that there is retention of a contextual threat conditioned HR response specific for the CTX+. There were no main effects or interactions of group (all Ps > 0.09).

To test for spontaneous recovery of conditioned threat responses HR, responses were subjected to phase (late extinction, early recovery test) x context (CTX+, CTX-) x group (R-Ext, Ext) 2x2x2 rmANOVA. There was an interaction effect of phase x context x time-point ($F_{3.277,150.760}$=3.710, p=0.011, η2=0.075), and an interaction of context x time-point ($F_{2.613,120.185}$=3.558 =, p=0.021, η2=0.072). We ran follow-up paired t-tests comparing HR in the CTX+ and CTX- for each time points separately for the late phase of extinction and the early phase of spontaneous recovery, but we did not find any differences. There were effects of group (all Ps > 0.3).

We also did not find any evidence for an effect of reinstatement on HR time courses. To test the effect of a reminder on reinstatement of contextual threat conditioned HR responses, HR time courses were subjected to phase (early, late reinstatement test) x context (CTX+, CTX-) x group (R-Ext, Ext) 2x2x2 rmANOVA. There were no effects (all Ps >0.169). To test for the increase in responses, HR time courses were subjected to phase (late recovery test, early reinstatement test) x context (CTX+, CTX-) x group (R-Ext, Ext) 2x2x2 rmANOVA. There were no effects (all Ps > 0.116).
